# Usability Evaluation of an Offline Electronic Data Capture App in a Prospective Multicenter Dementia Registry (digiDEM Bayern): Mixed Method Study

**DOI:** 10.2196/31649

**Published:** 2021-11-03

**Authors:** Michael Reichold, Miriam Heß, Peter Kolominsky-Rabas, Elmar Gräßel, Hans-Ulrich Prokosch

**Affiliations:** 1 Department of Medical Informatics, Biometrics and Epidemiology Friedrich-Alexander-Universität Erlangen-Nürnberg (FAU) Erlangen Germany; 2 Interdisciplinary Center for Health Technology Assessment and Public Health (IZPH) Friedrich-Alexander-Universität Erlangen-Nürnberg (FAU) Erlangen Germany; 3 Center for Health Services Research in Medicine Department of Psychiatry and Psychotherapy University Hospital Erlangen Erlangen Germany

**Keywords:** dementia, usability, evaluation, mobile device, registry, electronic data collection, offline, mobile app, digital health, usability testing

## Abstract

**Background:**

Digital registries have been shown to provide an efficient way of gaining a better understanding of the clinical complexity and long-term progression of diseases. The paperless method of electronic data capture (EDC) during a patient interview saves both time and resources. In the prospective multicenter project “Digital Dementia Registry Bavaria (digiDEM Bayern),” interviews are also performed on site in rural areas with unreliable internet connectivity. It must be ensured that EDC can still be performed in such a context and that there is no need to fall back on paper-based questionnaires. In addition to a web-based data collection solution, the EDC system REDCap (Research Electronic Data Capture) offers the option to collect data offline via an app and to synchronize it afterward.

**Objective:**

The aim of this study was to evaluate the usability of the REDCap app as an offline EDC option for a lay user group and to examine the necessary technology acceptance of using mobile devices for data collection. The feasibility of the app-based offline data collection in the digiDEM Bayern dementia registry project was then evaluated before going live.

**Methods:**

An exploratory mixed method design was employed in the form of an on-site usability test with the “Thinking Aloud” method combined with an online questionnaire including the System Usability Scale (SUS). The acceptance of mobile devices for data collection was surveyed based on five categories of the technology acceptance model.

**Results:**

Using the “Thinking Aloud” method, usability issues were identified and solutions were accordingly derived. Evaluation of the REDCap app resulted in a SUS score of 74, which represents “good” usability. After evaluating the technology acceptance questionnaire, it can be concluded that the lay user group is open to mobile devices as interview tools.

**Conclusions:**

The usability evaluation results show that a lay user group generally agree that data collecting partners in the digiDEM project can handle the REDCap app well. The usability evaluation provided statements about positive aspects and could also identify usability issues relating to the REDCap app. In addition, the current technology acceptance in the sample showed that heterogeneous groups of different ages with diverse experiences in handling mobile devices are also ready for the use of app-based EDC systems. Based on these results, it can be assumed that the offline use of an app-based EDC system on mobile devices is a viable solution for collecting data in a decentralized registry–based research project.

## Introduction

Patient registries have proven to be valuable tools for public health surveillance and research studies [1]. In these organized databases, observational study methods are used to collect uniform data and to evaluate specified outcomes for a defined population [2]. As an alternative to paper-based data collection, electronic data capture (EDC) systems have become established in registries in recent years [3]. In particular, EDC systems with web-based data collection tools have been widely accepted as the state-of-the-art approach in multicenter studies [4,5]. Such systems offer several advantages, including time and cost savings [6,7] or higher data quality [8-10] such as through plausibility checks during data entry. However, to make the most of these benefits, the EDC system must be designed for usability and tailored to the user’s preferred method of data collection [11].

To successfully integrate an EDC system into a registry-based research project, it is essential to coordinate the system requirements and usability with future users in advance [12,13]. Therefore, the usability of a system is critical to the success of interactive applications in health care [11]. Only a system that users see as fit for purpose has a better chance of being accepted and used in the long term [14,15]. In the fields of eHealth [16,17] and mobile health [18-20], user-centered usability studies are widely used to identify problems in systems. Although Schmier et al [21] already demonstrated the practical applications of usability theory for EDC systems in clinical trials in 2005, usability studies in EDC-based registry research are still comparatively rare [22].

The project “Digital Dementia Registry Bavaria (digiDEM Bayern)” [23] aims to establish a multicenter, prospective, longitudinal dementia registry. The data collection process is organized with a decentralized approach, including staff from different types of outpatient counseling and caring institutions distributed across Bavaria (Germany). Data collection often takes place in rural areas in the homes of people with dementia, where internet access is patchy or unavailable. In these cases, the project will gather electronic data offline using mobile devices and the app of the EDC system known as REDCap (Research Electronic Data Capture) [24,25].

Thus, the objectives of this study were to evaluate the usability of the REDCap app as an option for offline electronic data collection and to examine whether the target user group has the necessary technology acceptance for data collection using mobile devices. This study should help to identify potential barriers and evaluate the feasibility of the REDCap app in a registry study with a large number of distributed nonexpert data collection partners and the need for offline on-site data collection.

## Methods

### Setting

To foster dementia research, the registry project digiDEM Bayern [23] collects data from people with mild cognitive impairment or mild-to-moderate dementia and their family caregivers over a period of 3 years throughout all seven administrative districts of Bavaria. The findings will help to improve the living conditions of people with dementia and their caregiving relatives, especially in rural areas of Bavaria.

In the digiDEM project, data collection for the registry is carried out by approximately 300 so-called “digiDEM partners,” who are employees (such as nursing assistants and home health aides) from, for example, community counseling bodies, memory clinics, daycare facilities, or outpatient care organizations distributed across Bavaria that counsel or care for people with dementia and family caregivers. During face-to-face surveys involving interviews of people with dementia and their caregivers, the digiDEM partners enter various types of information [23], including data about diagnosis, cognitive trajectories, behavioral and psychological symptoms, and the care situation, into a web-based EDC system (REDCap). However, owing to the lack of mobility or poor health of people with dementia [26,27], conducting the survey at the digiDEM partner’s facility is not always possible. In these cases, the data must be gathered in the homes of people with dementia. Given the fact that access to the internet in Germany decreases with age (64.4% at age 73-78 years, 39.4% at age 79-84 years) and when people are living in rural areas [28,29], there is no guarantee that the digiDEM partners can use an existing on-site internet connection for the web-based EDC system while undertaking the survey at participants’ homes.

### System Description

In digiDEM, we use REDCap as our EDC system [24]. REDCap is a secure, web-based software platform designed to support data capture for research studies. In addition to the direct web-based data collection, REDCap offers the option of collecting data offline via the REDCap mobile app. The data can be synchronized to the central registry database subsequently [30]. REDCap has been adopted by more than 5265 partners in 142 countries since its initial development at Vanderbilt University [31].

In our usability evaluation, the app was used “out of the box” as offered by REDCap. The rendering style of the app was retained at the default setting “New render form.” Other than the current German language pack “German (DE)” being activated, no further adjustments were made to the REDCap app. The app is provided in English by default; however, there is an option to activate a language file to translate the user interface. Unfortunately, the German language files do not yet cover a full user interface translation (for some screenshots, see Multimedia Appendix 1). Therefore, some system messages are still in English, such as the synchronization report or error messages caused by missing values for mandatory fields. The questionnaire and project’s own customized warning messages for plausibility checks can be created in the REDCap designer function and are displayed in German.

### Material

Participants were provided with a tablet (Apple iPad Air 2 with iOS 13.7) to carry out the predefined tasks. The REDCap app (version 5.9.6) was preinstalled on the tablet and a dummy registry project with the test questionnaire for the usability study was set up. A user account was set up in advance for the participants. The test questionnaire contains a subset of questions from the original digiDEM questionnaire [23]. When designing the questionnaire, care was taken to use all field types that had also been used in the digiDEM questionnaire (textbox, drop-down list, radio buttons, multiple-choice checkboxes, date field). Some questions were linked by means of branching logic only to appear if the previous question was answered with a defined value. A plausibility warning message was also included to monitor the participants’ reactions to such warnings.

### Study Design

We performed a mixed methods study in our usability evaluation with an exploratory sequential design [32]. The qualitatively driven study (known as “Quant->qual”) started with a “Thinking Aloud” [33] component, followed by the standardized quantitative System Usability Scale (SUS) usability questionnaire. To also examine participants’ attitudes toward the mobile device–based data collection, a questionnaire based on the technology acceptance model (TAM) was applied. The study was performed over 4 weeks in October 2020.

Using a mixed methods approach, we were able to triangulate several data sources and hence consider the research question from different perspectives [34]: participants’ thoughts, demographic data, and structured questionnaires. Combining data from qualitative and quantitative methods can balance the strengths and offset possible limitations of the respective method [17], provide a more comprehensive understanding of evidence [35], and help researchers investigate the usability as comprehensively as possible [36,37]*.*

### Test Procedure

#### Design

**Figure 1:**
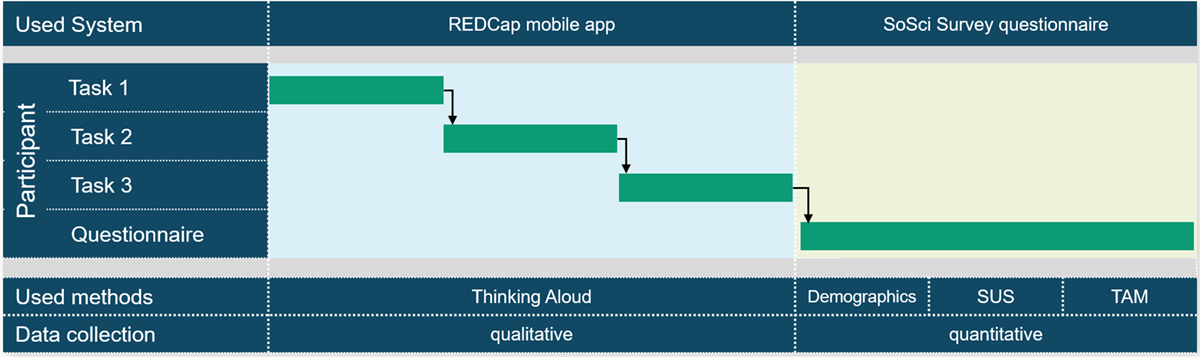
Overview of the systems and methods used during the test procedure. SUS: System Usability Scale; TAM: technology acceptance model; REDCap: Research Electronic Data Capture.

The test procedure consisted of two sequential independent parts, as illustrated in Figure 1. In the first part, qualitative data were collected using the Thinking Aloud method. In the second part, quantitative data were obtained using an online questionnaire.

We pretested the test procedure with four scientific project members to determine its suitability for obtaining rich data to address the proposed research objectives. Furthermore, technical and operational problems were addressed so that they could be excluded during the test.

Before starting the test procedure, the participants were informed about the app’s purpose and the test procedure. A demonstration video was produced in advance and shown before the test to familiarize participants with the Thinking Aloud method. In addition, a test manual was created, including all questions (for the participant) and answers (for the simulated patient) for the tasks in the test survey (Multimedia Appendix 2). To ensure that participants had sufficient information to perform the tasks, they received a brief in-person tutorial on using the REDCap app (Multimedia Appendix 3).

#### Qualitative Data Collection

Quantitative usability questionnaires cannot provide precise information about why a participant has rated usability in a particular way, and no direct usability problems can be derived from these responses [37]. Therefore, we used the Thinking Aloud method as a user-centered design approach to derive qualitative statements [38]. There is a potential risk of the participant forgetting to simultaneously express their thoughts while solving a task [39]. If a researcher actively intervenes and asks them to explain their thoughts, this can distract the participant’s attention or modify their thought processes. This interference can be seen as a kind of experimenter bias, which can strongly impact the reliability and validity of the qualitative data [40,41]. Therefore, the research team ensured as little interference as possible within the Thinking Aloud process [11]. Participants were simply reminded to keep talking if they stopped verbalizing their thoughts. Other, more intrusive types of probes to gather even more useful information were not used.

For the Thinking Aloud test, a digiDEM on-site interview situation was simulated. The participant had to enter the data into the app while interviewing people with dementia. The interviewee was simulated by a research assistant and was the same person for all participants. In the test survey, the participant had to complete three predefined tasks in the REDCap app. The tasks increased in complexity and represented realistic examples of tasks in the digiDEM data collection pathway. The first task required offline data collection in the form of a baseline interview, which included questions such as “What is your marital status?” or “Is there a medically confirmed diagnosis of dementia?” (Multimedia Appendix 1). The participant then had to upload the offline collected data to the server (synchronization) in the second task. In the final task, the participant conducted a follow-up interview.

#### Quantitative Data Collection

After completing the three tasks, the participant had to fill out an online questionnaire by means of the SoSci Survey tool [42], which consisted of sociodemographic data, the SUS [43], and a detailed TAM questionnaire (Multimedia Appendix 4).

The sociodemographic part included three closed questions on age, gender, and experience with technical devices. The SUS is a standardized scoring questionnaire that ensures a valid and reliable measurement for usability [44,45]. It provides usable results even with a smaller sample [46]. The German version of the SUS questionnaire was used in this study [47].

To rule out the possibility that a dismissive attitude toward mobile devices for data collection leads to poor usability, we evaluated technology acceptance based on the TAM. According to Davis [48], two factors are crucial for determining technology acceptance: perceived usefulness and perceived ease of use. Although the TAM is a well-established instrument for determining technology acceptance in health care [49], according to Holden and Karsh [50] and Ammenwerth [51], the model could benefit from modifications by taking into account external influencing variables in the health care environment. To account for this possibility, we included three further categories. The category “anxiety” was added [52] since some users experience anxiety when they are asked to use a new system [53]. The second category added was “social influence” [54]; this was added because digiDEM partners often work together in teams at their institution, and therefore the social environment could have an impact on acceptance [55,56]. Third, since the successful use of technology depends heavily on adequate organizational and technical infrastructure and support, the category “facilitating conditions” was also included [57]. Therefore, our TAM questionnaire contained five categories (perceived usefulness, perceived ease of use, anxiety, social influence, and facilitating conditions), each evaluated on a 5-point Likert scale. Each category was investigated by way of multiple items (Multimedia Appendix 4).

### Participants and Recruitment

We took care that the sociodemographic characteristics of the sample were as broadly distributed as possible to guarantee validity and trustworthiness of the qualitative data [58,59]. In particular, all age groups needed to be included in the sample, as age can significantly affect the usability rating [60,61]. For the sample selection, the digiDEM partners were first divided into subgroups based on the type of facility in which they worked: community counseling bodies, support groups, flat-sharing communities, daycare facilities, outpatient care organizations, geriatric rehabilitation facilities, and research institutes. From each subgroup, 1-2 participants were recruited randomly using balanced randomization.

We estimated requiring a minimum sample size of 12 participants based on our test procedure for a proper usability evaluation. Because the Thinking Aloud method provides a rich source of qualitative data [11], even a small sample (approximately 8 participants) is sufficient to understand the task behavior [62] and to identify the main usability problems [63]. For quantitative data, Tullis and Stetson [46] observed that even with 12 participants, the SUS questionnaire produced the same results as a larger sample in at least 90% of the cases studied.

### Inclusion and Exclusion Criteria

Participants in the study were selected from digiDEM partners who will eventually carry out the data collection as part of the digiDEM project. There were no limitations based on age, profession, or experience with information technology. Because digiDEM partners who have already gained experience with the REDCap app or another EDC system had to be excluded, we did not include memory clinic facilities that had participated in an earlier study.

### Data Analysis

#### Qualitative Data Analysis

The qualitative evaluation of the Thinking Aloud results was based on content analysis. Therefore, the participants were filmed while performing the tasks and a screen capture video of the tablet was recorded. All recordings of the Thinking Aloud test were transcribed verbatim and analyzed according to the structured content analysis method developed by Mayring [64]. The software MAXQDA (Version 2020 Plus) was used to transcribe the recordings and analyze data.

To ensure the trustworthiness of the qualitative data, we followed the checklist drawn up by Elo et al [65]. Accordingly, we started with a preliminary content analysis after the first participants had completed the test procedure. Two researchers (MR, MH) independently coded four interviews. To increase reliability, coders differed in age, gender, and professional background [64,66]. The participants’ statements were divided into the following main categories: “positive aspects,” “suggestions for improvement,” and “problems.” In line with the Zapf taxonomy of errors, the category “problems” was subdivided into either “functionality problems” or “usability problems” [67]. Functionality problems refer to the mismatch between the task being carried out and the app (eg, an error occurring while data are being uploaded), whereas usability problems refer to the mismatch between the user and app (eg, the app does not fit the user’s expectation because the logout button is only found on the landing page) [68,69].

Subcategories for the main categories were defined during the analysis. This category system served as the basis for coding the remaining transcripts. Given the descriptive nature of the data, additional subcategories emerged during coding. This process continued until saturation of the category system was achieved [58]. In a second run, a complete back check of the designed structure was performed. Finally, to increase trustworthiness, the entire research team reviewed the analysis process and categorizations in terms of researcher triangulation [70]. Differences in the coding were discussed and resolved by mutual agreement [71].

Because issues in the category “usability problems” mainly influence software usability, these statements were weighted by two independent researchers (MR, MH) according to the severity rating formulated by Nielsen [62,63]. The severity rating scale ranges from 0 to 4, where 0 means “I don’t agree that this is a usability problem at all” and 4 means “Usability catastrophe: imperative to fix this before product can be released” [62]. Major issues should be given higher priority because they can impact data quality, satisfaction, and functionality [22]. As Nielsen did not specify a method for calculating an overall severity score, we calculated an issue’s severity score by multiplying the severity rating by the number of mentions.

#### Quantitative Data Analysis

The SUS was evaluated using Brooke’s evaluation scheme [43]. Therefore, the weighting of the answers (1=strongly disagree to 5=strongly agree) was recoded. For positively worded questions, the most negative answer option (strongly disagree) was weighted with a 0 and the most positive answer (strongly agree) was weighted with a 4. For negatively worded questions, the coding was the exact reverse. All values per subject were summed and multiplied by 2.5. Thus, a value ranging from 0 (worst imaginable usability) to 100 (best imaginable usability) was achieved, ensuring the values’ comparability [43]. The boxplot form was chosen to present the data distribution based on the results of the TAM questionnaire in a standardized manner. All statistical calculations were performed with SPSS (Version 26.0) software.

### Ethical Statement

Before this study, approval was obtained from the institutional review board, the Committee on Research Ethics of the Friedrich-Alexander-University Erlangen-Nürnberg (Germany), following all applicable regulations (346_20 Bc). Informed consent was obtained in writing from all participants beforehand. Participation in the study was voluntary and no incentives were offered for participating.

## Results

### Participant Characteristics

In total, 12 participants took part in our usability study (6 men and 6 women). The participants were distributed over different types of institutions: community counseling (2), support groups (1), flat-sharing communities (1), daycare facilities (2), outpatient care organizations (2), geriatric rehabilitation facilities (2), and research institutes (2). The age of the participants covered all five age groups: 18-24 years, 25-34 years, 35-44 years, 45-49 years, and >60 years (Table 1).

When asked about experience with mobile devices (personal or professional), 10 out of 12 participants mentioned experience with a smartphone, half of the sample had experience using tablets, and one participant had no experience with any of the listed devices (Table 1).

None of the participants had any experience in EDC systems or registry-based research studies. These were medical assistants, nursing assistants, home health aides, and volunteer assistants, with caring for or counseling people with dementia and family caregivers as their primary role.

**Table 1:** Characteristics of the participants: age group, gender, and device experience.

Characteristics		Women (n=6), n	Men (n=6), n	Total (N=12), n
**Age group (years)**				
	18-24	1	0	1
	25-34	2	2	4
	35-44	0	1	1
	45-59	2	3	5
	>60	1	0	1
**Device experience (private or professional)**				
	Smartphone	6	4	10
	Tablet	2	4	6
	Desktop-PC	4	4	8
	Laptop	6	3	9
	None	0	1	1

### Thinking Aloud Test

#### Overview

The time it took participants to complete the three tasks in the REDCap app varied from 19 to 27 minutes. The coding of the Thinking Aloud method transcripts resulted in a total of 160 statements coded, including 44 positive aspects, 57 suggestions for improvement, 50 usability problems, and 9 functionality problems (all statements were counted individually). The coded transcript can be found in Multimedia Appendix 5.

#### Positive Aspects

A total of 44 positive statements could be identified. As shown in Table 2, these were categorized into six subcategories (grouped by participants).

Seven out of 12 participants were optimistic about the app’s learnability and navigation: “Once you’ve done it a couple of times, you’re good with it” or “At the beginning, I was a bit confused...but now I understand it better already.” One research partner described the first steps from logging in to collecting data as “so even for me so far, foolproof.” Another positive aspect was the app’s feedback function for the project’s own built-in plausibility checks and warning messages: “Yes, that is also very helpful in any case, that you get the error message right away.” Feedback from the system such as receiving green symbols after successful saving of the questionnaires gave the participants a sense of security: “Yes and now it’s green. So, I assume that everything is saved.” In addition, some participants described the app as “clearly designed” and “well structured.”

**Table 2:** Distribution of the subcategories for “positive aspects.”

Subcategory (positive aspects)	Participants, n
Learnability	7
Navigation	7
Feedback	6
Instructions	5
Design	4
Structure	3

#### Functionality Problems

Two functionality problems were identified that were seen as most important. The first occurred while synchronizing the data collected offline. Two participants were unable to transfer the data. One participant commented: “‘Stop sending modified records to server’ [warning message in REDCap]—okay, let’s see what’s wrong. This should not happen, right?” Because the detailed description of the problem was in English, the participants could not solve it themselves. The problem arose because no data synchronization had occurred before the data collection. Therefore, the project in the app must be synchronized before the offline survey is carried out to prevent an interruption. The second problem became evident in the third task. The participant was supposed to conduct a follow-up interview in the record of the baseline interview (task 1). However, the participant created a new record and collected the follow-up interview data for a new study participant. The REDCap app should not offer this option as it requires further action to link the data to the existing data set from the baseline interview.

#### Usability Problems

Special attention was paid to the category “usability problems,” mainly influencing the app’s usability. A total of 50 statements were categorized into 6 subcategories. Table 3 shows the usability problems, sorted by the severity score, which resulted from multiplying the severity rating by the number of mentions.

**Figure 2:**
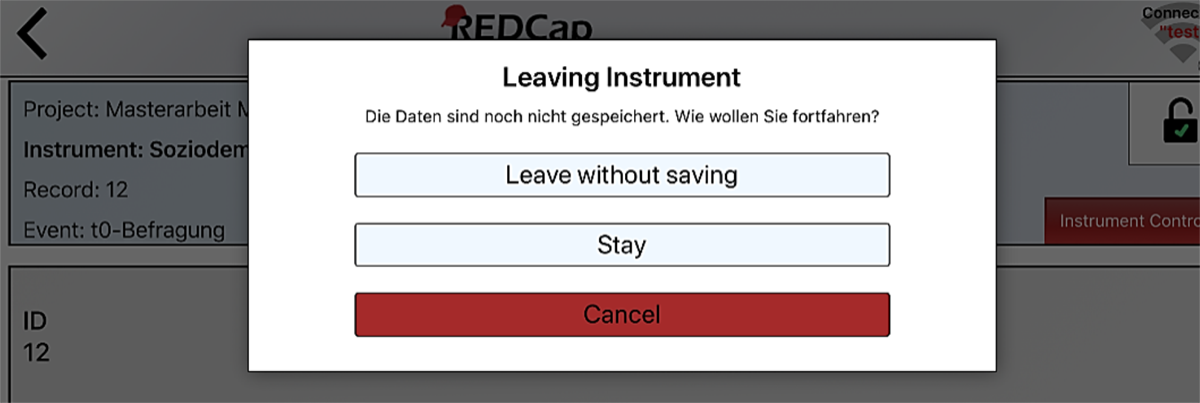
Screenshot of the REDCap app with a mix of German and English.

The REDCap app offers the option of translating the app’s interface using a language file. Nevertheless, not all terms have yet been translated into German. Some error and warning messages from the system are still in English, such as the message that appears when a user leaves a questionnaire without first saving it (Figure 2).

**Table 3:** Categories of usability problems and the derived importance discovered in the test.

Usability problem	Severity rating	Number of mentions	Severity score
Language	3	6	18
Feedback	3	4	12
Perceived offer character	2	6	12
Inconsistent interaction design	2	4	8
Navigation	1	7	7
Knowledge error	1	5	5

One participant was critical of the increased time required to interpret the English language: “I must ensure that I manage my time well and therefore I can’t constantly think about what that means.” The mixing of German and English bothered two users and resulted in some confusion: “It’s still a bit confusing. A few times it’s in German, then again in English. You have to switch quickly in your mind.” It is noticeable that the language barrier was complained about by participants from the two youngest age groups, who also achieved a lower SUS score.

The lack of information given to users about what is happening in the app (“feedback”) led to confusion during the survey and delayed task processing: “So here I’m not sure how to proceed.” This also led to uncertainty such as with regard to whether or not the synchronization task was successful: “For me, it’s unfortunately not apparent whether the data has been uploaded or not.” It is noteworthy that this usability problem only affected the participants who had no experience using tablets.

Participants were sometimes unaware of a function behind an interactive element such as a button (“perceived offer character”). For example, after logging into the app, the user must actively select a project to collect data, even if the user had been authorized only for one project. One participant described this situation as follows: “Next I go to ‘My Projects’ [4 second pause] Okay, the button’s missing, or I’m really having a blackout.” Especially in the older age group (45-59 years), buttons not being recognized as functions was a recurring problem.

**Figure 3:**
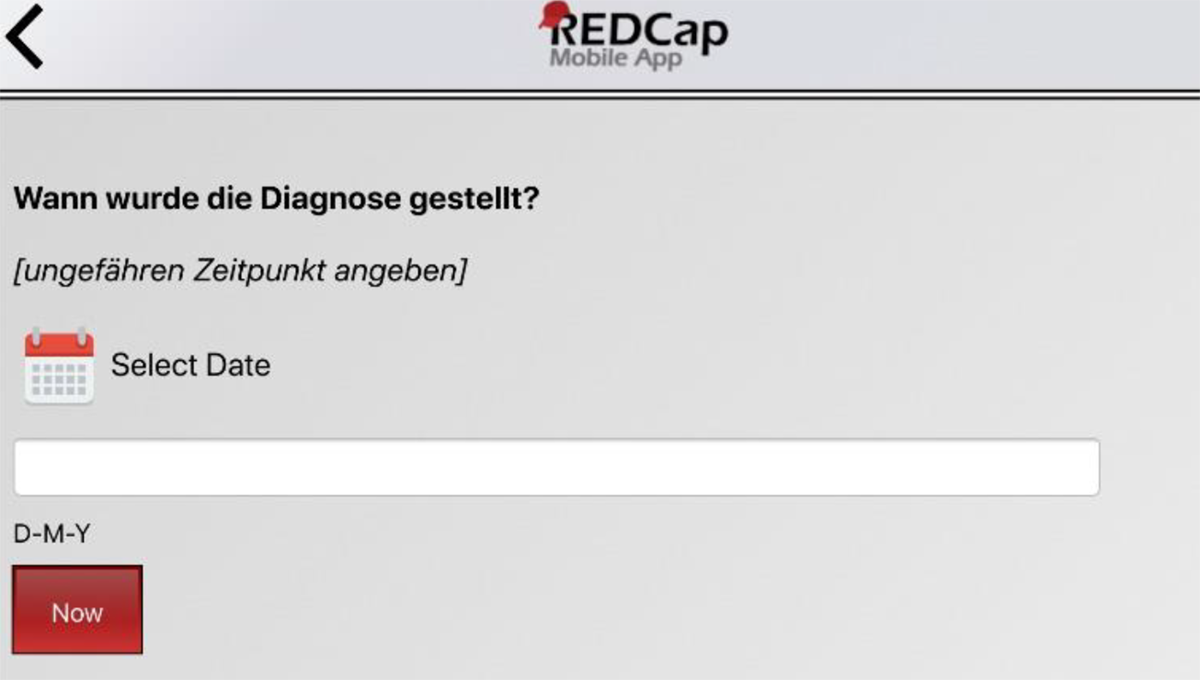
Screenshot (in English and German) of a question with the field type "date".

In some situations, the participants expected a different function based on the design (“inconsistent interaction design”). The field type “date” was especially challenging for four participants. As shown in Figure 3, the “date” field offers both a calendar icon and a textbox. However, the date could only be selected by clicking on the calendar icon. Four participants needed several attempts to select the correct date because the date field behaved differently from previous input fields.

Although many participants appreciated the navigation in the app, they felt some navigation procedures to be too complicated (“navigation”). In particular, the pathway to logging out of the app was felt to be too cumbersome to find: “So how do I get out of here now?” Some usability problems were also caused by knowledge gaps (“knowledge error”): “How do I see what survey date I’ve chosen?”

#### Suggestions for Improvement

Some participants immediately provided suggestions for improvement after pointing out a problem. A total of 57 suggestions for improvement were identified, which were categorized into 5 subcategories (grouped by participants). Eight out of 12 participants suggested more explicit feedback, especially when synchronizing data during the second task: “Just a confirmation, for example, a pop-up message ‘Date successfully transferred,’ that I know I can go back.” Seven participants requested a “notes” field to collect additional data: “I would find it helpful to have the option to make notes quickly.” REDCap offers the function “field notes,” but it was not intuitive for the participants to find. Furthermore, 4 of the 8 participants would have found it more intuitive if they could have gone directly to the next question using the enter key: “For me, it would be helpful that it then jumps to the next question.” In addition, 5 out of 12 participants suggested an easier way to log out: “Simple ‘logout’ would have been clearer.” Four participants proposed a more flexible option for language selection, either to switch entirely to German or to have more language options for colleagues whose native language is neither German nor English: “I also have colleagues who speak German but come from another country. So maybe another translation is necessary.” Moreover, 3 out of the 12 participants suggested more color tones and graphic highlighting in the design. Another participant would have preferred an indication of the progress of data collection: “for example, ‘You have completed 18 of 20 survey forms’.”

### Identified Implications and Recommendations

Based on the usability problems found and suggestions for improvement, we identified implications and recommendations (Table 4). While analyzing the video and screen recordings, we observed that many of the usability problems led to uncertainty among participants, also resulting in delays in the survey process. In addition, this may cause data to be partially collected, or in the worst case, an interview could be cut short.

There are short-term solutions that the project team can provide, such as targeted user training for the identified issues or providing a test environment for users to familiarize themselves with the system. There is also long-term optimization potential that should be addressed by REDCap’s developers, such as including a user expertise-based help option within the app.

**Table 4:** Identified problems of the usability test, and short- or long-term solutions.

Problem		Short-term solution (by the project)	Long-term optimization (by the developers)
**Usability problems**			
	Language	User training; provide a document with translations and explanations; check and adjust existing REDCap language file	Simplified language selection; complete translation, including system messages and synchronization report
	Feedback	User training; provide a test system	User expertise-based help
	Perceived offer character	User training; provide a test system	Optimization of user interface
	Inconsistent interaction design	User training; provide a test system	Enable input by typing or hide input field; provide keyboard type based on the field type (eg, numeric keyboard)
	Navigation	User training; create a short paper-based how-to guide	Simplification of navigation (eg, log out)
	Knowledge error	User training; provide filling out instructions directly in the questionnaire	User expertise-based help
**Functionality problems**			
	Data synchronization	User training; include a note at the end of the questionnaire to regularly synchronize the records	Sending notifications on the device in case of nonsynchronized records
	Follow-up interview	User training; create a short paper-based how-to guide for follow-up interviews	Optimized visit overview

### SUS Questionnaire

The overall SUS score of the REDCap app was 74. According to the Bangor classification, this represents “good usability” [44]. The SUS scores for each participant are shown in Table 5. The rows represent the recoded data of individual SUS questions and the columns refer to the individual participants. The highest value was 90 and the lowest value was 55. Participants from the oldest and the second-youngest age groups rated the app with “excellent” usability (SUS=90). Participants with experience using a tablet achieved an average SUS score of 75.8, compared to 72.9 for participants who did not have tablet experience.

The majority of participants indicated that they would use the REDCap app frequently (8 of 12 participants). Furthermore, 8 participants found the app easy to use. Ten out of 12 participants could imagine that most people can quickly learn to use REDCap. Concerning the need for technical assistance in using the app, the results indicated good usability, as 10 of the 12 respondents disagreed or strongly disagreed that help was needed. Nevertheless, half of the participants felt the system was unnecessarily complex and 5 participants disagreed with the statement about feeling confident using the app. None of the negatively worded questions received a “strongly agree” rating.

**Table 5:** Detailed System Usability Scale (SUS) scores for each participant (N=12).

SUS item	Participant number (age group, years)													Mean (SD)		Total		SUS score
	P1 (25-34)	P2 (18-24)	P3 (45-59)	P4 (45-59)	P5 (45-59)	P6 (25-34)	P7 (25-34)	P8 (35-44)	P9 (25-34)	P10 (45-59)	P11 (>60)	P12 (45-59)						
I think that I would like to use this system frequently	2	2	3	2	3	3	4	2	3	3	4	4	2.91 (0.79)		35.0		87.5	
I found the system unnecessarily complex	2	3	2	1	2	3	4	4	4	1	4	3	2.75 (1.13)		33.0		82.5	
I thought the system was easy to use	3	2	4	2	1	4	3	2	3	3	3	3	2.75 (0.86)		33.0		82.5	
I think that I would need the support of a technical person to be able to use this system	3	3	4	3	1	4	4	3	3	1	4	3	3.00 (1.04)		36.0		90.0	
I found the various functions in this system were well integrated	3	2	4	2	4	3	3	2	4	3	3	4	3.08 (0.79)		37.0		92.5	
I thought there was too much inconsistency in this system	2	4	2	4	2	3	3	3	4	1	3	3	2.83 (0.75)		34.0		85.0	
I would imagine that most people would learn to use this system very quickly	3	3	4	4	3	4	3	2	2	4	3	4	3.25 (0.75)		39.0		97.5	
I found the system very cumbersome to use	2	3	4	3	2	4	3	3	4	4	4	4	3.33 (0.77)		40.0		100	
I felt very confident using the system	2	1	3	1	2	4	3	2	3	4	4	3	2.66 (1.07)		32.0		80.0	
I needed to learn a lot of things before I could get going with this system	2	3	2	4	2	4	3	3	3	4	4	4	3.16 (0.83)		38.0		95.0	
Total	24	26	32	26	22	36	33	26	33	28	36	35	N/A^a^		N/A		N/A	
SUS score	60	65	80	65	55	90	82.5	65	82.5	70	90	87.5	N/A		N/A		N/A	

^a^N/A: not applicable.

### Technology Acceptance Questionnaire

**Figure 4:**
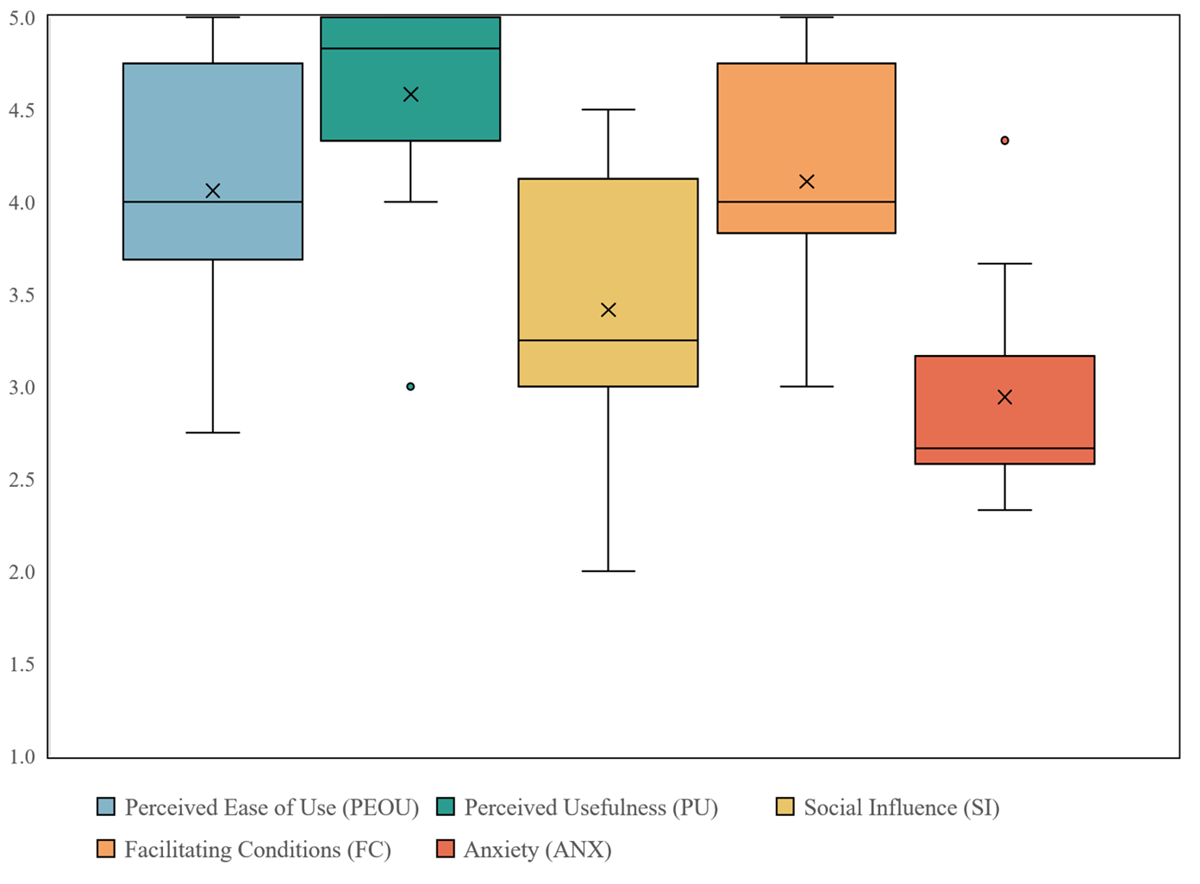
Distribution of the Technology Acceptance Model categories.

An extended TAM was used to evaluate how the participants accept mobile devices for data collection. From a maximum 75-point scale (15 questions multiplied by the highest response value of 5), the participants scored an average of 60.5 points (SD 7.3). The number of items collected in the questionnaire was reduced to the following underlying factors that determine the items’ average scale value: perceived ease of use, perceived usefulness, social influence, facilitating conditions, and anxiety. The boxplot in Figure 4 shows the distribution of the technology acceptance categories. The perceived usefulness of mobile devices was rated as very high by participants (mean 4.58), indicating that they consider mobile devices to be helpful in their work, as these enable faster processing of tasks and make work easier. The category anxiety met with the lowest approval (mean 2.94), which indicates that the participants have little concern about using mobile devices and are not afraid to use them for their work. The largest heterogeneity in responses (SD 0.82) was found in the category social influence, which implies that the participants tend to receive different recommendations on the use of mobile devices from their work colleagues and that some supervisors support the use of mobile devices while others do not.

## Discussion

### Principal Results

The success of a registry depends on the quality of the data collected. Most registry studies use pilot testing to evaluate the correct implementation of the questionnaires used in the EDC system. A usability evaluation of whether users can cope with the EDC system in the intended environment is often ignored. This is even more important because usability problems can affect whether an app is ultimately adopted or abandoned [11,72].

Most of the digiDEM partners had not yet had any experience in registry research. They are therefore considered to be “lay users,” as they are neither familiar with registry research studies nor using an app on a mobile device for data collection. Conventional EDC systems are intended to be used by professional registry research staff at a clinic site [22]. In addition, there are numerous differences between web-based and app-based data collection that can affect usability, such as smaller displays with adjusted interfaces and different input devices on mobile devices or different workflows due to data synchronization. In longitudinal studies such as digiDEM Bayern, it is essential to adhere as closely as possible to the specified survey dates to guarantee the study’s timeliness and validity [21]. To avoid waiting until a patient returns to a particular facility or environment, offline on-site data collection via a mobile app provides a valuable tool for more effective decentralized registry studies.

Our usability evaluation helped us to identify issues that could affect the usability of offline data collection with the REDCap app (Table 4). With the help of the participants’ thought processes during the Thinking Aloud test, it was possible to gain a more nuanced understanding of participants’ behaviors, intentions, and expectations. Therefore, we were able to uncover usability issues even after participants had successfully completed a task. Participants also made suggestions for improvement to increase efficiency and data quality. The three most significant usability issues were the language barrier, lack of feedback, and unperceived nature of the procedures the app offered. Although these are not critical problems, they do limit usability.

The language by which a system communicates with a user can have a major influence on usability [22,73]. For the efficient use of an EDC system, inexperienced users should especially be able to use their native language. The language could affect the acceptance of a system, as shown in other studies [74,75]. Participants’ statements in our study confirmed this. In contrast to English system messages, messages in German (eg, those arising from self-designed plausibility checks) were perceived as positive by the participants. Bearing this in mind, a complete translation of the app should lead to an increased SUS score (ie, a higher rating of the usability of the REDCap app).

In their study about the impact of usability on software design, Juristo et al [73] found further usability issues that were also addressed by our participants, such as “feedback.” Insufficient feedback from the REDCap app, identified as a usability issue by some participants, made participants feel insecure.

All field types used in the questionnaire should be tested, especially when using nontext-based items such as date selection fields or visual scales [76]. To ensure that users are able to complete the study questionnaire, these nontext-based items must be as usable on the tablet as regular text-based items.

The data synchronization process was identified as a major functionality usability problem. Uploading the data collected offline was also noted as problematic in a study of implementation strategies for the REDCap app by McIntosh et al [77]. The developers of REDCap acknowledge [30]: “Sending data is, at present, a complicated process.” Since the app does not automatically upload the collected records as soon as an internet connection is available, the user must actively start the process. This can lead to unsynced data and data loss in the worst case. Therefore, the process and its importance must be explicitly explained and trained.

Among the positive aspects of the qualitative usability evaluation, learnability was particularly highlighted. The participants who were not experienced in handling mobile devices became more familiar with the app and the tablet from task to task. The results of the quantitative SUS questionnaire confirmed the positive statements. For example, learnability was also one of the highest-rated items in the SUS. Given the widespread use of the SUS, a comparison with existing SUS study results is possible [78]. With a SUS score of 74, the REDCap app’s usability is above the average SUS score of 68, which was calculated from the results of around 500 studies [44].

Good usability cannot always predict the likelihood of future use, as other factors may also play a role. For example, anxiety can lead to a system being perceived as not easy to use, even if it has been designed to be user-friendly [54]. It is essential to ensure that future users accept an app on a mobile device for data collection before introducing such technology. In addition to the costs involved, less user acceptance of the technology can lead to less frequent use [79] and lower performance [80]. For these reasons, eliciting feedback from data collectors is essential to avoid jeopardizing success in a registry project. Considering that many studies have problems reaching their target number of participants [81,82], a lack of technology acceptance or usability issues should not additionally lead to lower numbers of participants. The results of our TAM questionnaire support the findings of the Thinking Aloud test and the SUS questionnaire that the REDCap app and tablets will be accepted and used by the user group examined here. The two categories “perceived usefulness” and “perceived ease of use” were rated highly. “Anxiety” can also be ruled out as an obstacle to using the technology. Only in the area of “social influence” would broader support be desirable. Here, the lack of digitization at some institutions is a likely variable [83,84].

By evaluating the usability and acceptance of app-based offline data collection at an early stage in our project, we were able to identify usability problems that need to be considered when introducing such a data collection method. As Qiu and Yu [85] have shown, the results of the Thinking Aloud test can be used not only to evaluate usability but also to determine the training needs of novice system users. We were able to confirm this with our findings (see Table 4). Based on the identified issues, individual training measures focusing on certain issues could be generated. Furthermore, setting up a test environment should help reduce participants’ feelings of insecurity before an initial survey.

### Comparison With Prior Work

The advantages of a system with good usability, such as enhanced efficiency and user acceptance, less training effort, or higher data quality, are indisputable [11,86]. However, usability evaluations are rarely used for EDC systems in clinical and registry research. Welker [87] identified numerous barriers and solutions during the implementation of EDC systems without explicitly addressing the usability issue. McIntosh et al [77] described an implementation strategy of mobile technologies for data collection using the REDCap app in survey research projects. The primary focus of their study was the technical feasibility of the offline data collection process rather than its usability. There are also guidance documents in place [88] or under development [89] that address the requirements and issues of EDC in clinical trials. However, usability is not, or only briefly, mentioned in these guidelines.

Only a few studies such as those by Ndlovu et al [90] and Dunn et al [91] have compared the usability of the EDC system REDCap to other data collection methods such as paper-based or Microsoft Excel/Access. Due to the limitation of using only a quantitative questionnaire, they could not identify specific usability issues in their studies. To our knowledge, there are no other studies that feature a usability evaluation of the REDCap mobile data collection using a qualitative-driven mixed methods approach. This also allowed us to identify specific usability problems.

Walden et al [22] recently took a different approach, suggesting that it is useful to perform a usability study while EDC tools are still being developed to improve the usability of such systems from the start. Nevertheless, the results of our work suggest that registry studies with already developed EDC systems can benefit from usability evaluations with respect to the individual registry requirements and the particular health care setting.

### Limitations

Three limitations of the study should be acknowledged. First, the sample size (N=12) could be seen as too small for meaningful assessments to be extrapolated. Even though larger samples are usually recommended for quantitative studies, Tullis and Stetson [46] and Nielsen and Landauer [92] have shown that the SUS questionnaire and usability tests can deliver meaningful results even with 12 participants. Furthermore, Nielsen [93] and Faulkner [94] confirmed that a small sample of test subjects is sufficient to apply the Thinking Aloud method as part of a usability test. Compared to other studies, similarly large sample sizes could provide valid results [95]. The sample size also depends on how many different platforms are evaluated [61]. Therefore, it can be assumed that 12 participants was a suitable sample size for this study.

Second, participation in the study was voluntary. In this case, only participants who had experience with mobile devices or technically interested participants can be presumed to have participated in the study. As shown by the participants’ characteristics (Table 1), only half the sample had experience using tablets. This ratio corresponds to the German average [96]. One participant had no mobile device experience at all. However, it cannot be ruled out that usability could be rated more highly in a sample of participants who are familiar with mobile devices.

Third, the usability testing was laboratory-based. We replicated a real scenario [97] for all participants by simulating the context and the interviewing of people with dementia. Simulating an interview partner is essential for systems that use branching logic, where the order of the questions is determined by the interview partner’s answer [98]. Nevertheless, it cannot be excluded that the app’s usability during actual interviews of people with dementia might differ from our results [21].

### Conclusions

Offline registry data collection can be made more efficient through EDC systems, but attention must be paid to the usability of these systems. Despite the widespread use of usability tests in the health care and app environment, usability evaluations in the field of electronic data collection in registry-based research have so far remained scarce. Our study shows that it is profitable to conduct a usability evaluation of the EDC system considering future users and the project environment. Using a mixed method approach, we identified positive and negative aspects regarding the usability of an EDC app for offline data collection. By addressing these aspects, the registry project digiDEM Bayern can avoid pitfalls and realize the benefits of EDC systems, even in areas where using web-based EDC systems is not viable due to unreliable internet connectivity. The out-of-the-box use of the REDCap app resulted in a good usability rating, which can be further improved by addressing the identified issues by means of user training of digiDEM partners and improvements on the part of REDCap’s developers. The technology acceptance in the sample showed that heterogeneous groups of different ages with varying experiences in handling mobile devices are open to the use of app-based EDC systems. Based on these results, it can be assumed that the offline use of an app-based EDC system on mobile devices is a viable solution for collecting data in a registry-based research project.

## Appendix

Multimedia Appendix 1 Test survey in the REDCap mobile app.

Multimedia Appendix 2 Test manual with tasks and answers for the test survey.

Multimedia Appendix 3 Introductory training to the REDCap mobile app.

Multimedia Appendix 4 Online questionnaire with sociodemographic data, System Usability Scale, and technology acceptance model.

Multimedia Appendix 5 Thinking Aloud results (encoded segments).

## Abbreviations

digiDEM Bayern: Digital Dementia Registry Bavaria
EDC: electronic data capture
REDCap: Research Electronic Data Capture
SUS: System Usability Scale
TAM: Technology Acceptance Model
