# Supplementary material for: Usability Evaluation of an Offline Electronic Data Capture App in a Prospective Multicenter Dementia Registry (digiDEM Bayern): Mixed Method Study
Source: JMIR Form Res. 2021 Nov 3;5(11):e31649. doi: 10.2196/31649 (PMC8600440; doi:10.2196/31649)

Supplementary Appendix 1

REDCap Test Survey

Content:

- Event: Baseline interview
  - Instrument: Sociodemographic data
  - Instrument: Diagnosis-specific data
  - Instrument: Media usage

- Event: Follow-up interview
  - Instrument: Diagnosis-specific data

- Example of how it was displayed on the tablet

Event: Baseline interview | Instrument: Sociodemographic data

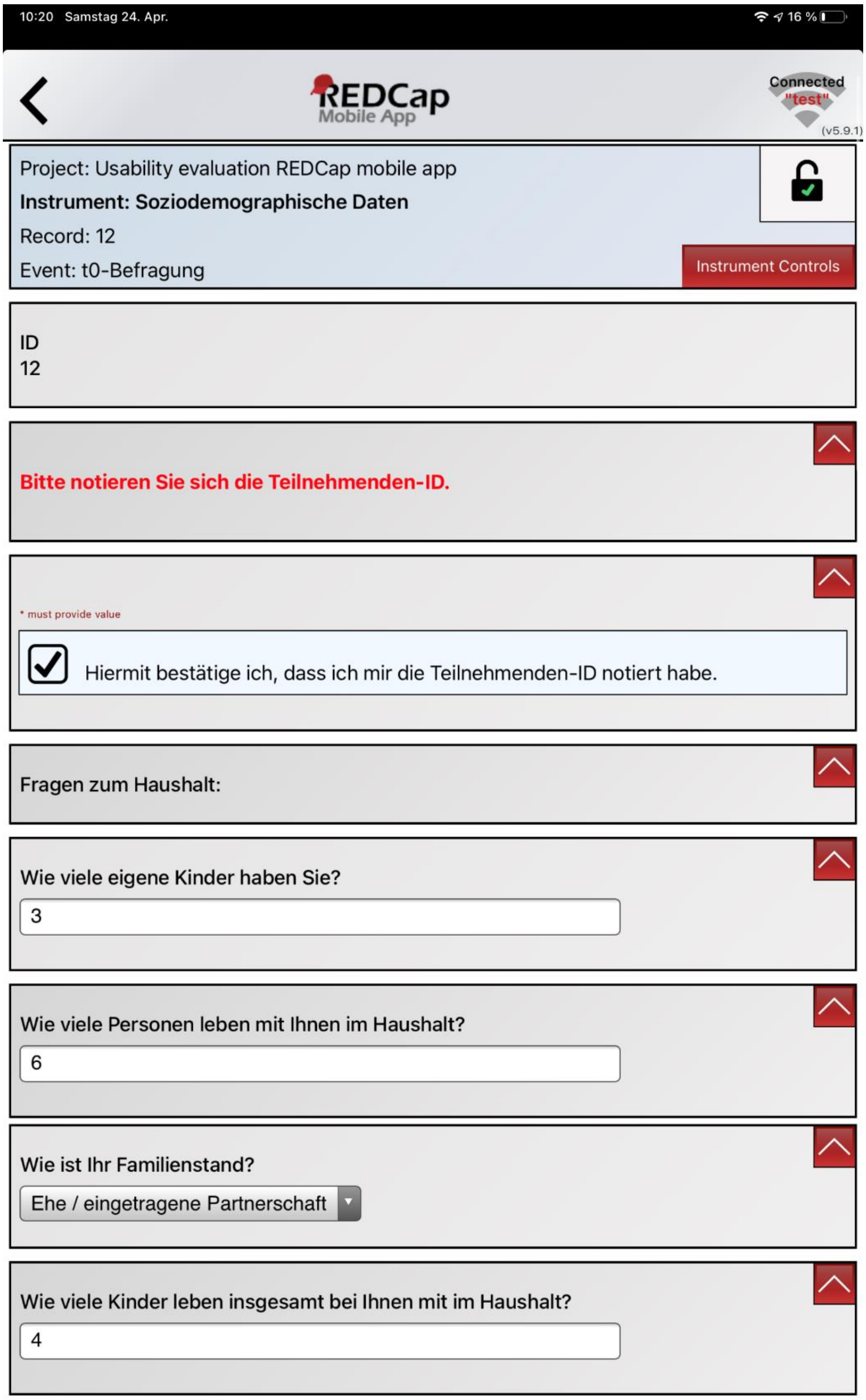

Wie ist Ihr Familienstand?

Ehe / eingetragene Partnerschaft

Wie viele Kinder leben insgesamt bei Ihnen mit im Haushalt?

4

Was ist Ihr höchster Schulabschluss?

- ( ) kein Schulabschluss
- ( ) Volksschule
- ( ) Hauptschule
- (•) Mittlere Reife
- ( ) Fachhochschulreife
- ( ) Abitur

Wie ist Ihre berufliche Situation? (Mehrfachantwort möglich)

- [ ] vollzeiterwerbstätig
- [ ] teilzeiterwerbstätig
- [x] geringfügig erwerbstätig (Minijob)
- [ ] RenterIn
- [ ] arbeitslos / arbeitssuchend
- [ ] erwerbsunfähig
- [x] Hausfrau / -mann
- [ ] sonstiges

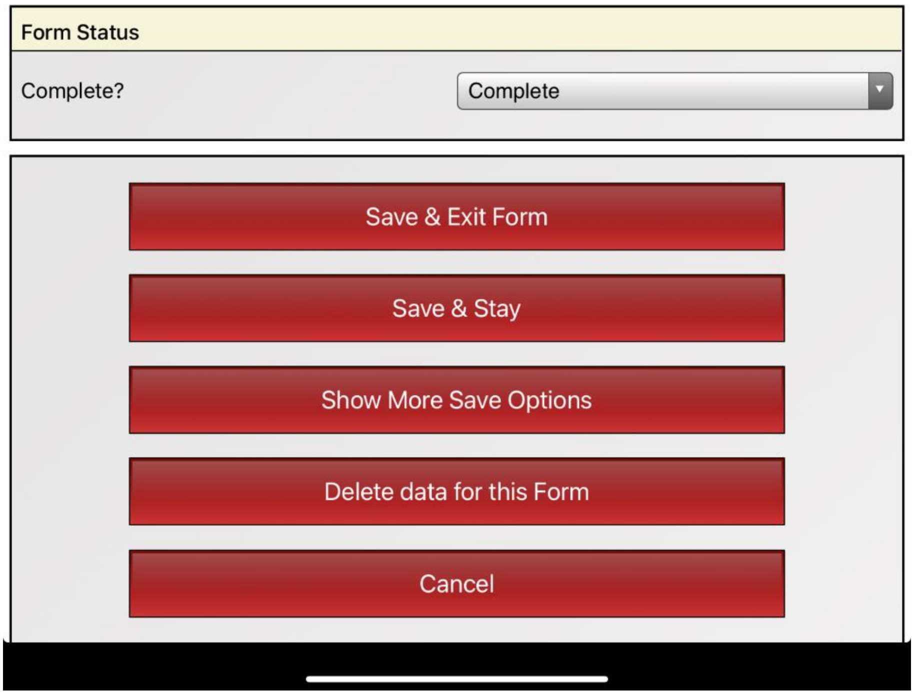

Event: Baseline interview | Instrument: Diagnosis-specific data

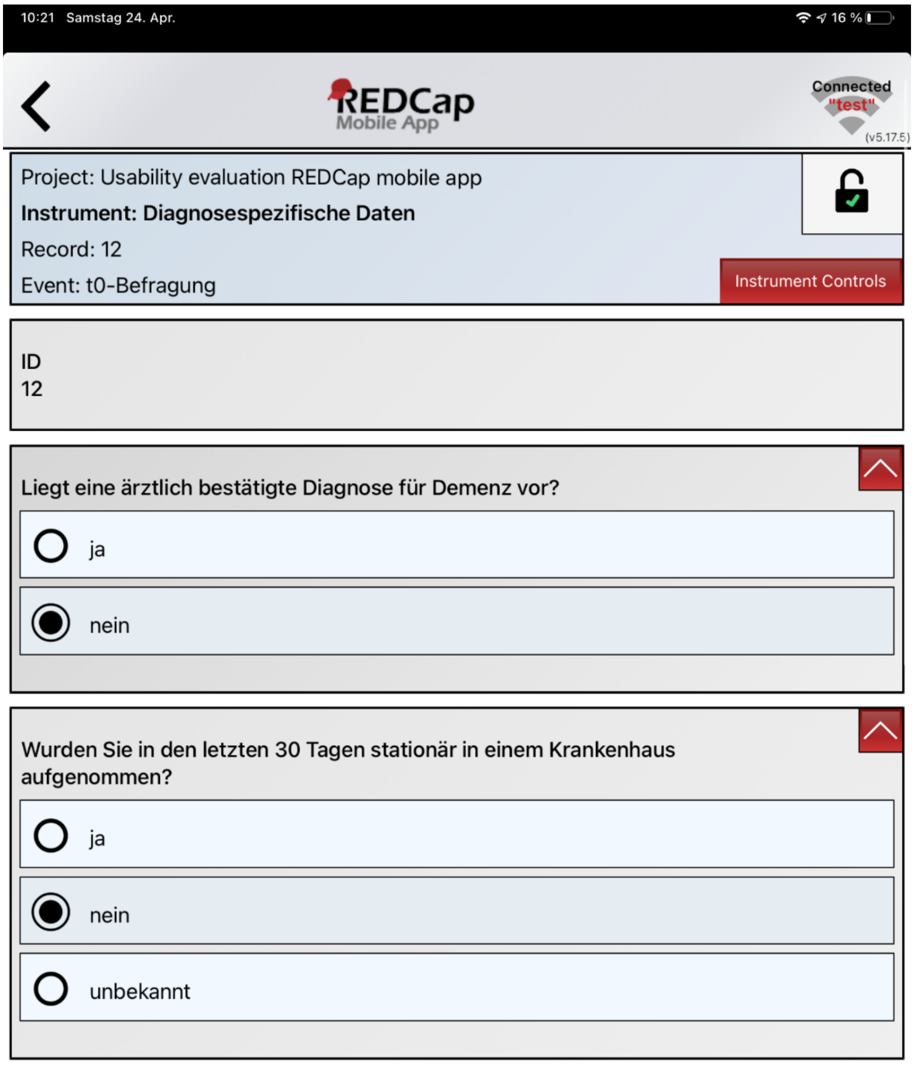

Waren Sie in den letzten 30 Tagen bei einem niedergelassenen Arzt in Behandlung?

- (●) ja
- ( ) nein

Wo fand die ambulante Untersuchung statt?

- [x] Hausarzt
- [ ] Zahnarzt
- [x] Facharzt
- [ ] Krankenhaus
- [ ] Rehabilitationseinrichtung

Form Status

Complete? Complete

Save & Exit Form

Save & Stay

Show More Save Options

Delete data for this Form

Cancel

Event: Baseline interview | Instrument: Media usage

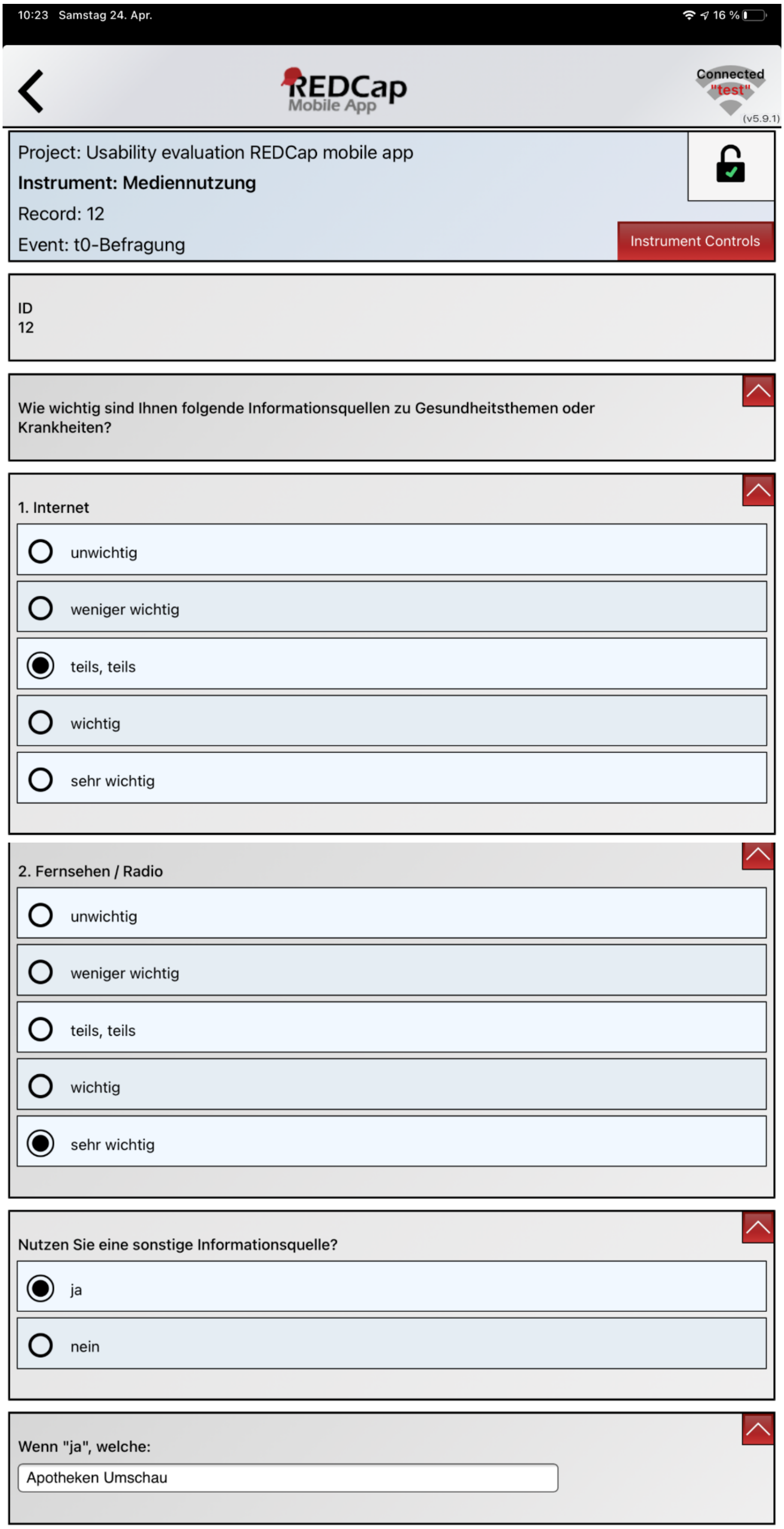

10:23 Samstag 24. Apr. 16 %

REDCap Mobile App

Connected "test" (v5.9.1)

Project: Usability evaluation REDCap mobile app

Instrument: Mediennutzung

Record: 12

Event: t0-Befragung

Instrument Controls

ID
12

Wie wichtig sind Ihnen folgende Informationsquellen zu Gesundheitsthemen oder Krankheiten?

1. Internet

- ○ unwichtig
- ○ weniger wichtig
- ● teils, teils
- ○ wichtig
- ○ sehr wichtig

2. Fernsehen / Radio

- ○ unwichtig
- ○ weniger wichtig
- ○ teils, teils
- ○ wichtig
- ● sehr wichtig

Nutzen Sie eine sonstige Informationsquelle?

- ● ja
- ○ nein

Wenn "ja", welche:

Apotheken Umschau

Wichtigkeit dieser Informationsquelle:

- ○ unwichtig
- ○ weniger wichtig
- ○ teils, teils
- ◉ wichtig
- ○ sehr wichtig

Internetnutzung

Wie oft haben Sie im Durchschnitt in den letzten 3 Monaten das Internet genutzt?

- ○ jeden Tag oder fast jeden Tag
- ○ mindestens ein Mal in der Woche
- ◉ weniger als ein Mal in der Woche

Für welche Zwecke haben Sie das Internet genutzt? (Mehrfachantwort möglich)

- [x] Kommunikation
- [x] Informationssuche
- [ ] Unterhaltung
- [ ] Gesundheit

Form Status

Complete? Complete

Save & Exit Form

Save & Stay

Show More Save Options

Delete data for this Form

Cancel

Event: Follow-up interview | Instrument: Diagnosis-specific data

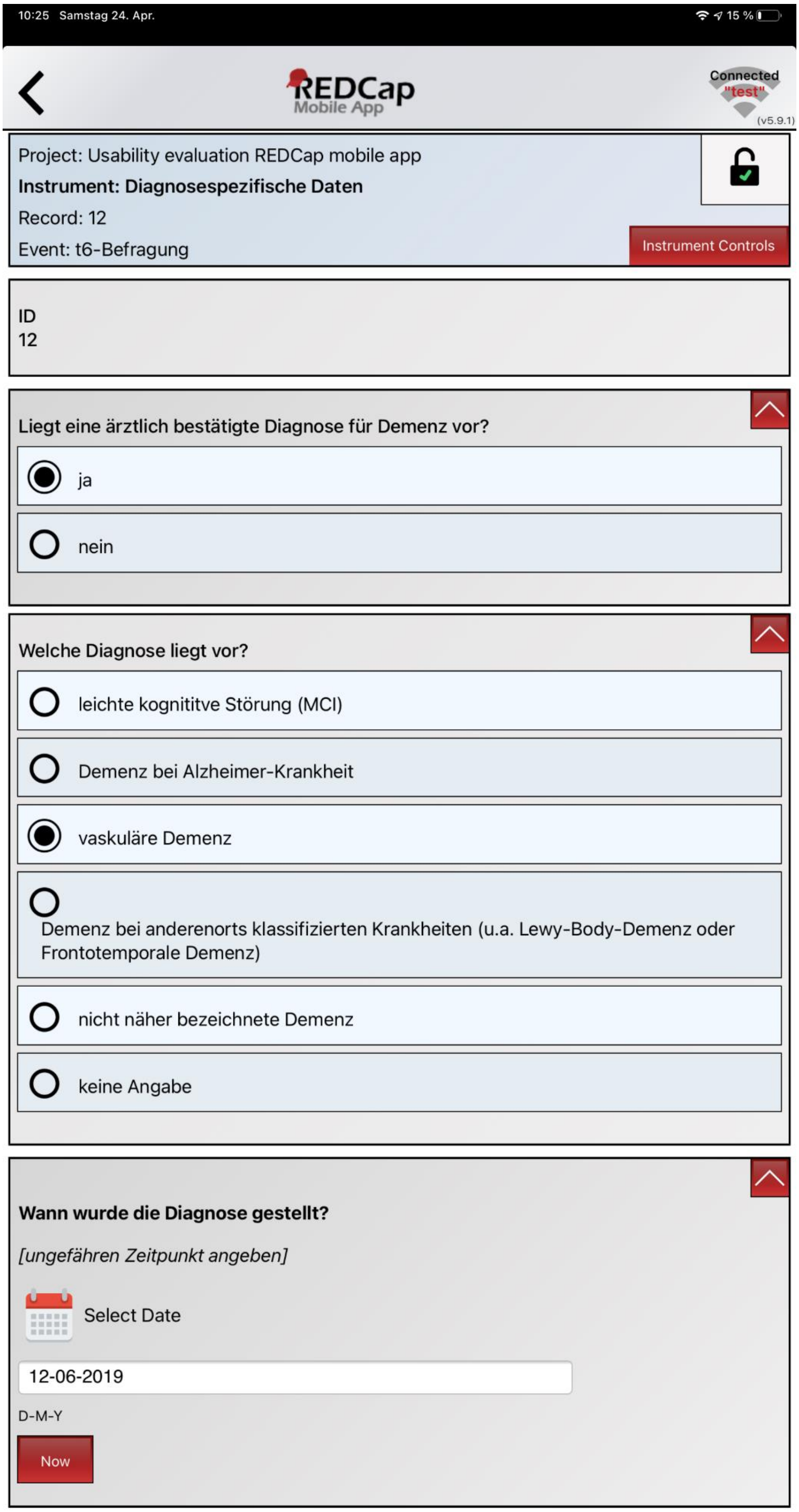

Wurden Sie in den letzten 30 Tagen stationär in einem Krankenhaus aufgenommen?

- (●) ja
- ( ) nein
- ( ) unbekannt

Wie oft mussten Sie stationär im Krankenhaus aufgenommen werden?

1

Wieviele Nächte haben Sie insgesamt im Krankenhaus verbracht?

10

Was war der Grund für die stationäre Behandlung im Krankenhaus?

Gestürzt

Waren Sie in den letzten 30 Tagen bei einem niedergelassenen Arzt in Behandlung?

- ( ) ja
- (●) nein

Form Status

Complete? Complete

Save & Exit Form

Save & Stay

Show More Save Options

Delete data for this Form

Cancel

Example of how it was displayed on the tablet

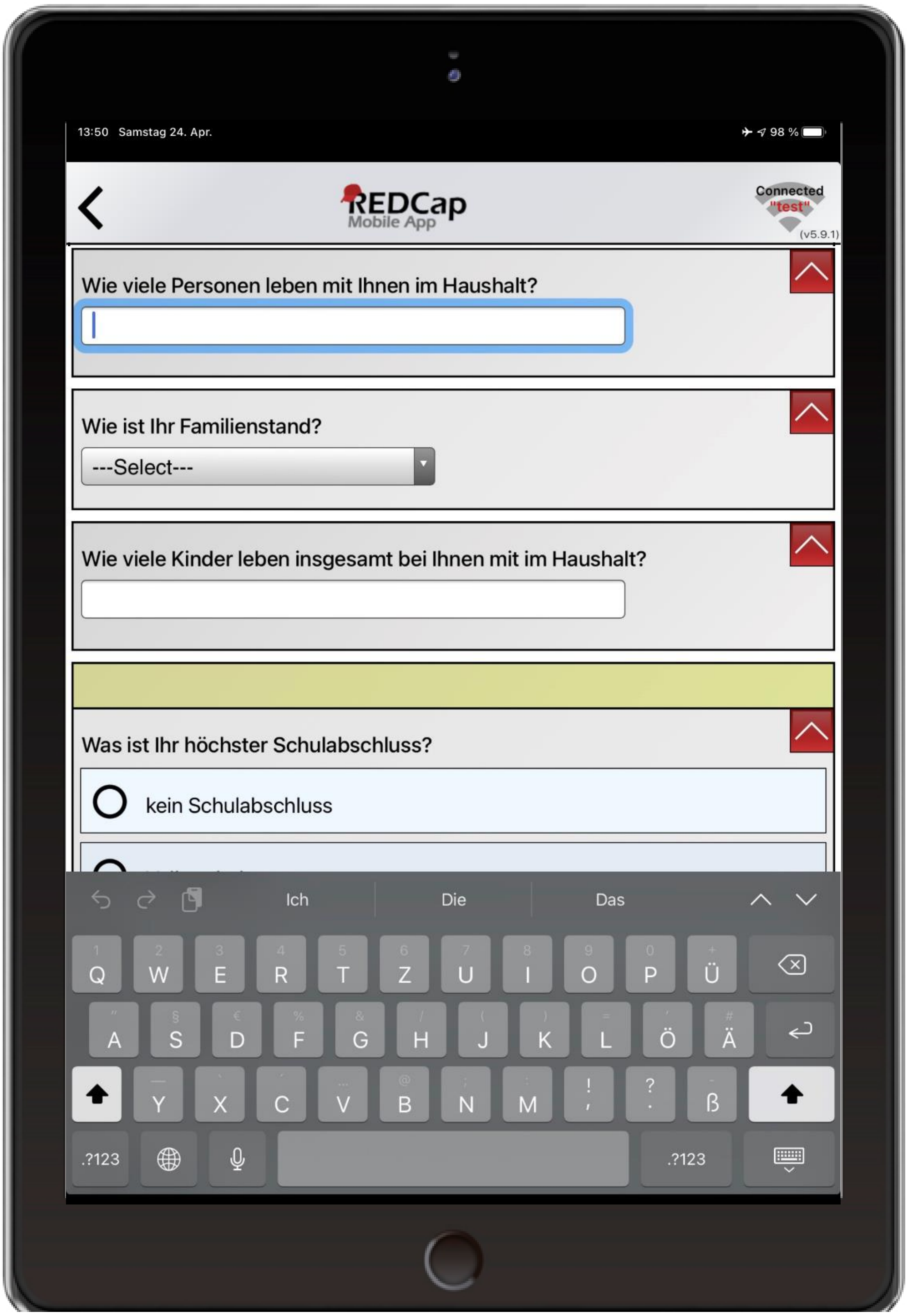

Supplement: Multimedia Appendix 1 [file formative_v5i11e31649_app1.pdf]
