# Supplementary material for: Usability Evaluation of an Offline Electronic Data Capture App in a Prospective Multicenter Dementia Registry (digiDEM Bayern): Mixed Method Study
Source: JMIR Form Res. 2021 Nov 3;5(11):e31649. doi: 10.2196/31649 (PMC8600440; doi:10.2196/31649)
Supplement: Multimedia Appendix 2 [file formative_v5i11e31649_app2.pdf]

Supplementary Appendix 2

Test manual

Content:

- Part 1 - REDCap Tasks for the participant of the usability evaluation
- Part 2 - Answers to the REDCap survey for the simulated patient

Note: For the usability evaluation questionnaire, the digiDEM questionnaire was shortened enormously, and individual questions were selected. The focus was on using every possible field type, e.g., date field, dropdown lists, radio buttons, and checkboxes. Furthermore, filter questions and logic checks with error messages were included, as they can also occur in the project.

Thinking Aloud Test

Thank you for participating in the usability test! Today you will test the data collection method for the digital dementia register digiDEM Bayern. Use the tablet and the pre-installed app REDCap to solve the selected tasks. One after the other, you will complete three tasks. Please take your time for each task.

Please think aloud during the whole processing and speak out what thoughts are going through your mind. Please comment continuously on your action. Feel free to praise as well as criticize the app.

Sample questions you can ask yourself during the Thinking Aloud test include:

- Was the app easy or difficult to use?
- What features of the app surprised you?
- Which features did you like, which did you not?
- What did you expect? Are there differences between what you expected and what you found?

Copyright: © 2020 Lehrstuhl für Medizinische Informatik

FRIEDRICH-ALEXANDER UNIVERSITÄT ERLANGEN-NÜRNBERG
Universitätsklinikum Erlangen
MEDICAL VALLEY
gefördert durch Bayerisches Staatsministerium für Gesundheit und Pflege

Task 1: Conduct baseline interview

Situation:

You are starting the "digiDEM Bayern" project at your facility. As part of the data collection, you have traveled to the home of your participant Max Maier. You do not have Internet connectivity on site. Nevertheless, you can conduct the interview because the REDCap app automatically saves the collected data offline on the tablet.

Your access data for the app are:

Username: test

PIN: 251661

What to do:

- Conduct a baseline interview (t0 interview) with Mr. Maier
- To do this, record the patient data in the survey project 'Usability evaluation REDCap mobile app' using the corresponding survey forms
- Save and exit each fully processed data entry form
- At the end of the baseline interview, check whether all survey forms are complete → lights up green (= complete)

Note:

As you complete the task, please continuously express aloud your thoughts regarding the usability of the app.

Copyright: © 2020 Lehrstuhl für Medizinische Informatik

gefördert durch Bayerisches Staatsministerium für Gesundheit und Pflege

Task 2: Transfer of the offline data

Situation:

You have arrived back at your institution and have an Internet connection. In order for the data stored on the tablet from Mr. Maier's t0 baseline interview to be successfully entered into the database, you must transfer the data collected offline to the system.

What to do:

- Send offline collected data to the system
- If all options light up green, you have successfully transmitted the data
- After that, you can close the app (Logout)

Note:

As you complete the task, please continuously express aloud your thoughts regarding the usability of the app.

Copyright: © 2020 Lehrstuhl für Medizinische Informatik

gefördert durch
Bayerisches Staatsministerium für
Gesundheit und Pflege

Task 3: Conduct follow-up interview

Situation:

For the follow-up interview six months later, Mr. Maier comes to your institution. Conduct the follow-up interview (t6) together with him.

What to do:

- Conduct a follow-up survey (t6 interview) with Mr. Maier
- To do this, record the patient data in the survey project 'Usability evaluation REDCap mobile app' using the corresponding survey forms
- Select the corresponding record of Mr. Maier from the baseline interview
- Save and exit each fully processed data entry form
- At the end of the follow-up survey, check whether all survey forms are complete
- After that, you can close the app (Logout)

Note:

As you complete the task, please continuously express aloud your thoughts regarding the usability of the app.

You have now successfully completed all tasks.

Thank you for your participation!

Copyright: © 2020 Lehrstuhl für Medizinische Informatik

FRIEDRICH-ALEXANDER UNIVERSITÄT ERLANGEN-NÜRNBERG
Universitätsklinikum Erlangen
MEDICAL VALLEY
gefördert durch Bayerisches Staatsministerium für Gesundheit und Pflege

Thinking Aloud Test - Answers

Task 1: Conduct baseline interview

Sociodemographic data

☐	Question 1: How many children of your own do you have?<br>➢ 3
☐	Question 2: How many people live with you in the household?<br>➢ 3<br>➢ Later correction: number of persons in the household → 6 (wife + husband + three own children + one child of the wife)
☐	Question 3: What is your family status?<br>➢ marriage / registered partnership
☐	Question 4: How many children in total live with you in the household?<br>➢ 4
☐	Question 5: What is your highest school degree?<br>➢ Intermediate maturity
☐	Question 6: What is your professional situation?<br>➢ houseman<br>➢ marginally employed (minijob)

Diagnosis-specific data

☐	Question 1: Is there a medically confirmed diagnosis of dementia?<br>➢ No
☐	Question 2: Have you been admitted to a hospital as an inpatient in the last 30 days?<br>➢ No

Copyright: © 2020 Lehrstuhl für Medizinische Informatik

☐	Question 3: Have you seen an outpatient doctor in the last 30 days?<br><br>➢ yes
☐	Question 4: Where did the outpatient examination take place?<br><br>➢ family doctor and specialist

Media usage

☐	Question 1: How important are the following sources of information on health topics or diseases to you?<br><br>➢ Internet: **fairly important**<br>➢ Television / Radio: **very important**
☐	Question 2: Do you use any other source of information?<br><br>➢ yes<br><br>If "yes," which ones:<br><br>➢ „Apotheken Umschau“<br><br>Importance of this source of information:<br><br>➢ important
☐	Question 3: On average, how often have you used the Internet in the last 3 months?<br><br>➢ less than once a week
☐	Question 4: For what purposes did you use the Internet?<br><br>➢ Communication<br>➢ Information search

Task 2: Transfer of the offline data

No answers are needed there.

Copyright: © 2020 Lehrstuhl für Medizinische Informatik

digiDEM BAYERN

Task 3: Conduct follow-up interview

Diagnosis-specific data

☐	Question 1: Is there a medically confirmed diagnosis of dementia?<br><br>➢ Yes, vascular dementia
☐	Question 2: When was the diagnosis given?<br><br>➢ 12.06.2019
☐	Question 3: Have you been admitted to a hospital as an inpatient in the last 30 days?<br><br>➢ Yes<br><br>How many times did you have to be admitted to the hospital as an inpatient in the last 30 days?<br><br>➢ Once<br><br>How many nights in total did you spend in the hospital?<br><br>➢ 10 nights<br><br>What was the reason for inpatient treatment in the hospital?<br><br>➢ Cause: fallen
☐	Question 4: Have you seen an outpatient doctor in the last 30 days?<br><br>➢ No

Copyright: © 2020 Lehrstuhl für Medizinische Informatik

gefördert durch Bayerisches Staatsministerium für Gesundheit und Pflege
