# Supplementary material for: Usability Evaluation of an Offline Electronic Data Capture App in a Prospective Multicenter Dementia Registry (digiDEM Bayern): Mixed Method Study
Source: JMIR Form Res. 2021 Nov 3;5(11):e31649. doi: 10.2196/31649 (PMC8600440; doi:10.2196/31649)
Supplement: Multimedia Appendix 3 [file formative_v5i11e31649_app3.pdf]

Supplementary Appendix 3

REDCap app introduction

Content:

- Induction to data collection with the REDCap app

Note: Before the participants started with the test survey, they received a short introduction to the data collection using the REDCap app. The mixture of English and German language can be seen in the screenshots, which were not explicitly translated.

Short briefing on data collection with the mobile application REDCap

digiDEM BAYERN

Structure

1. Log in REDCap
2. Selection of the projecct
3. Create a new survey
4. Save data entry form
5. Data transfer
6. Important terms

digiDEM BAYERN

Login | Select project | New participant | Save data | Data transfer | Important terms

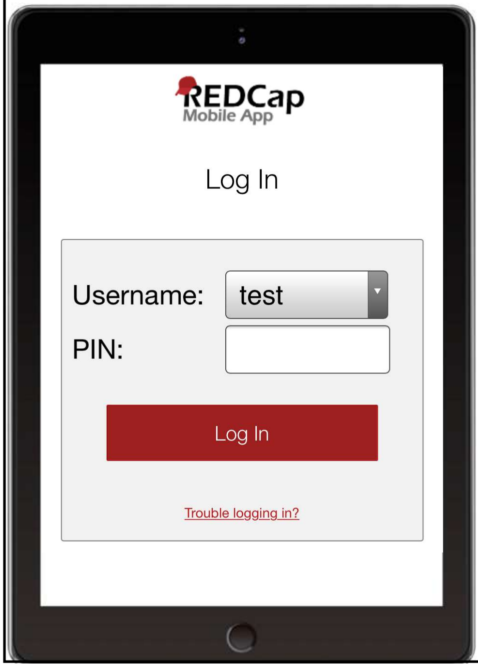

- Please log in with the following user data:
- Username: test
- PIN: 251661

digiDEM BAYERN

Login | Select project | New participant | Save data | Data transfer | Important terms

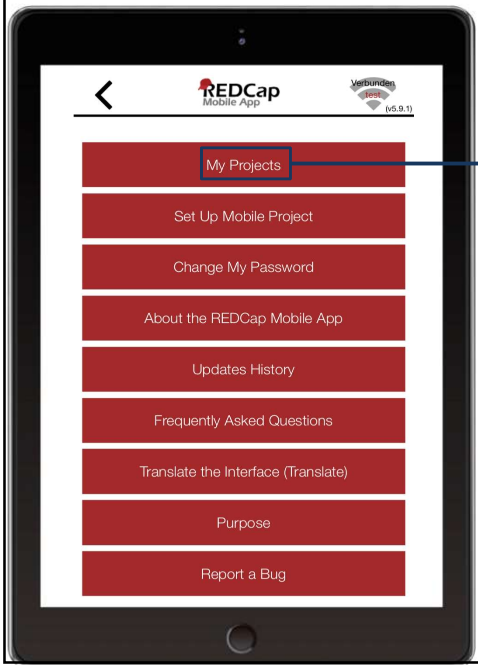

Select ‘My Projects‘ to go to the survey project

digiDEM BAYERN

Login | Select project | New participant | Save data | Data transfer | Important terms

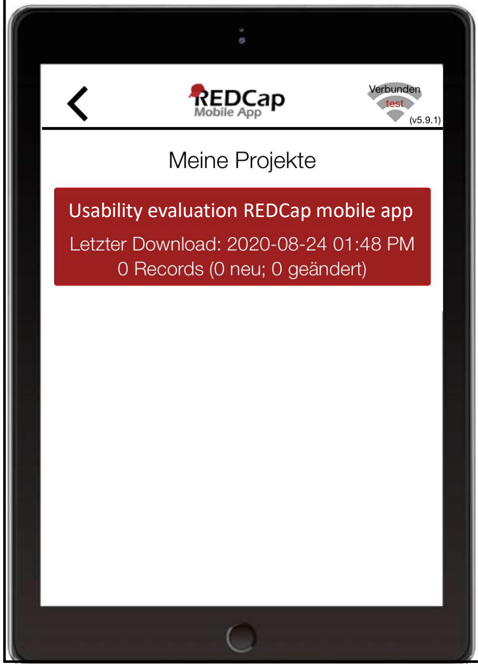

- Select survey project ‚Usability evaluation REDCap mobile app‘

digiDEM BAYERN

Login | Select project | New participant | Save data | Data transfer | Important terms

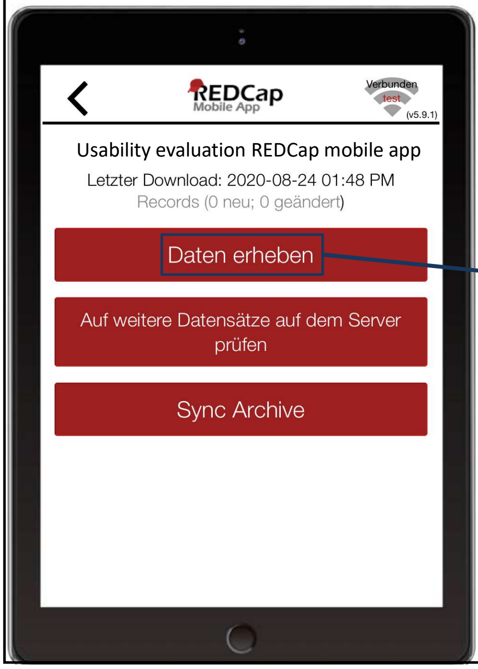

To be able to collect patient data, select ‘Collect data’

digiDEM BAYERN

Login | Select project | **New participant** | Save data | Data transfer | Important terms

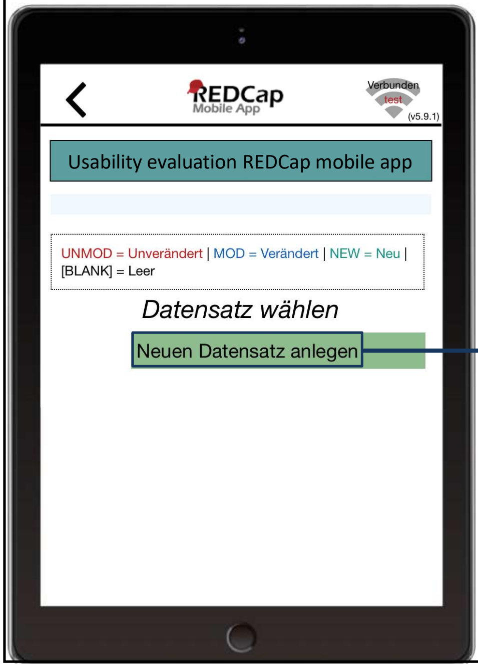

- A data record is created for each new patient
- One data record is used per patient

digiDEM BAYERN

Login | Select project | **New participant** | Save data | Data transfer | Important terms

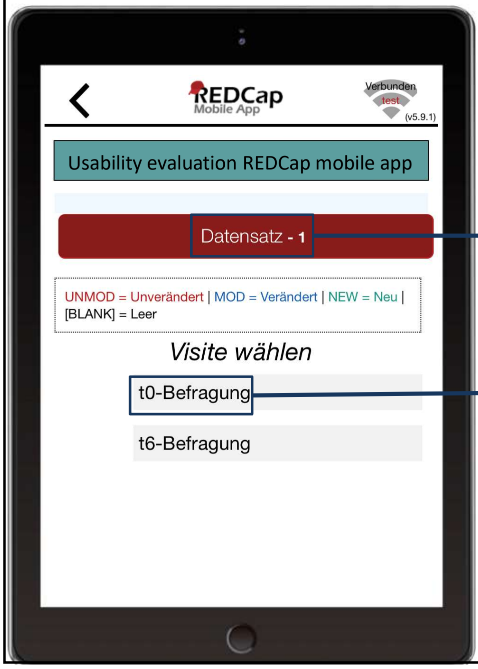

Data record - 1
(= Participant-ID Nr. 1)

Select time of interview

digiDEM BAYERN

Login | Select project | **New participant** | Save data | Data transfer | Important terms

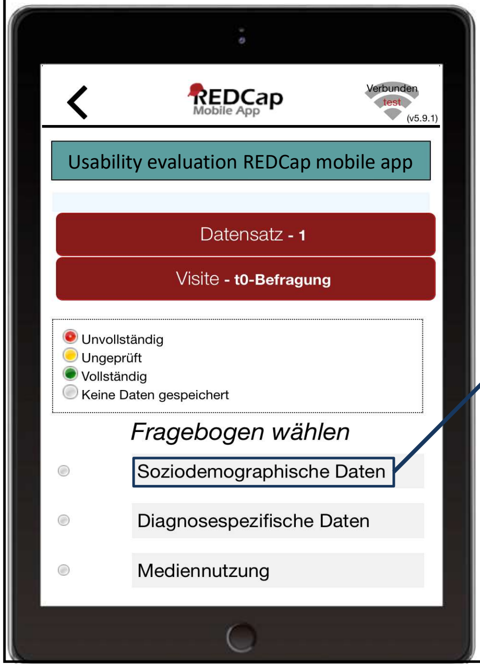

- The respective data of the patient with the participant ID no. 1 at baseline interview t0 can be recorded
- t0 interview consists of the three forms ‘Sociodemographic data’, ‘Diagnosis-specific data’ and ‘Media use’.
- Start with ‘Sociodemographic data’ survey form

digiDEM BAYERN

Login | Select project | Neue Befragung | **Save data** | Data transfer | Important terms

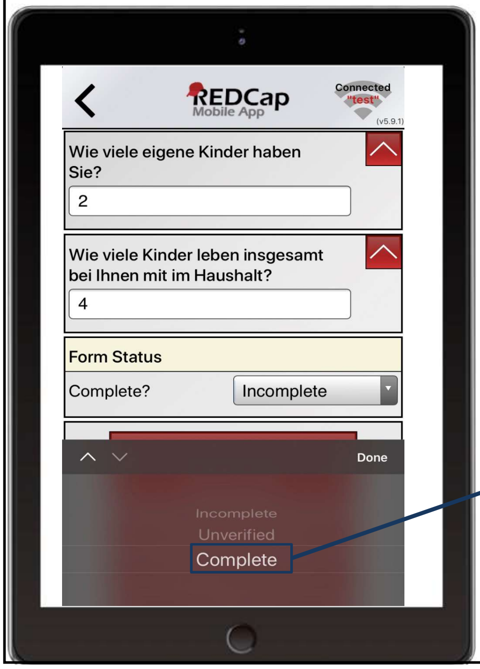

Status of a completely filled out data entry form is set to ‘Complete’

digiDEM BAYERN

Login | Select project | New participant | **Save data** | Data transfer | Important terms

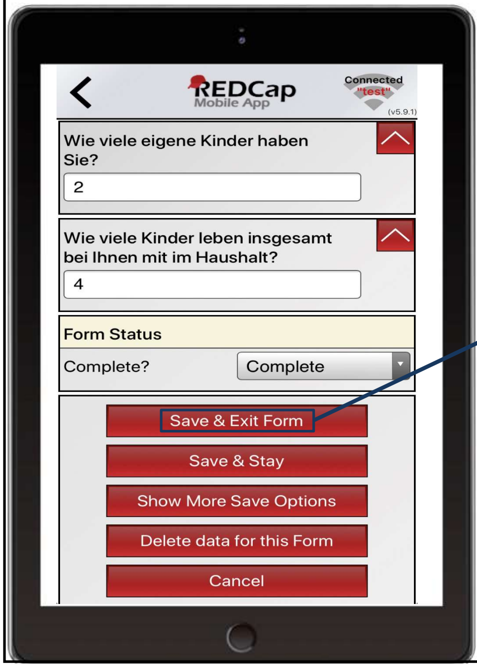

Save and exit data entry form

- Recording of sociodemographic data of the patient with participant ID 1 at baseline interview t0 is completed

digiDEM BAYERN

Login | Select project | New participant | Save data | **Data transfer** | Important terms

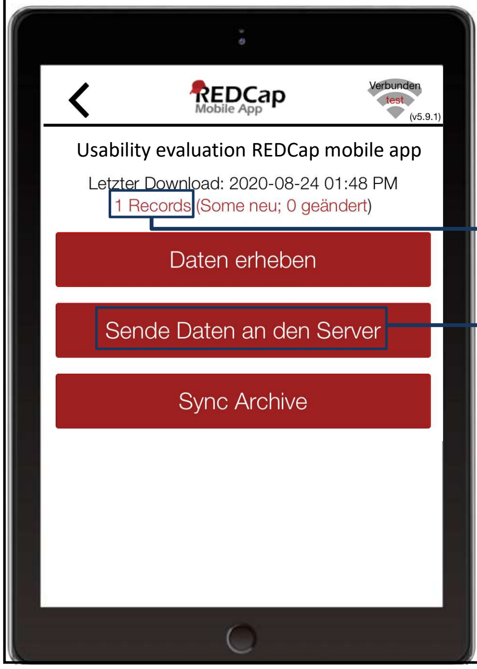

Data collection was performed offline

Transfer data to the server

digiDEM BAYERN

Login | Select project | New participant | Daten speichern | **Data transfer** | Important terms

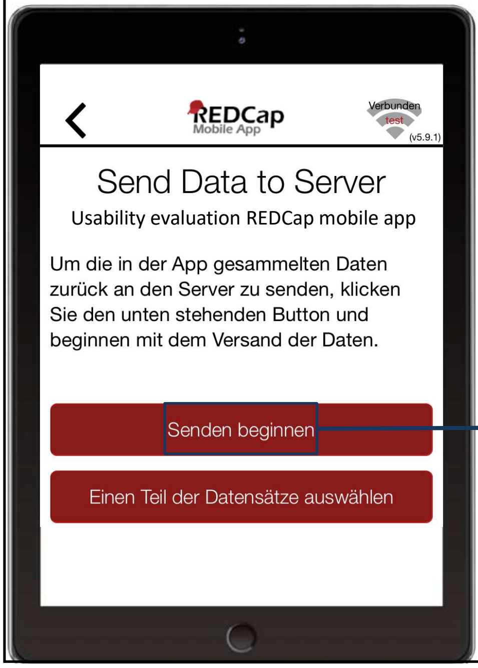

Data transfer through ‚Start sending'

digiDEM BAYERN

Login | Select project | New participant | Save data | **Data transfer** | Important terms

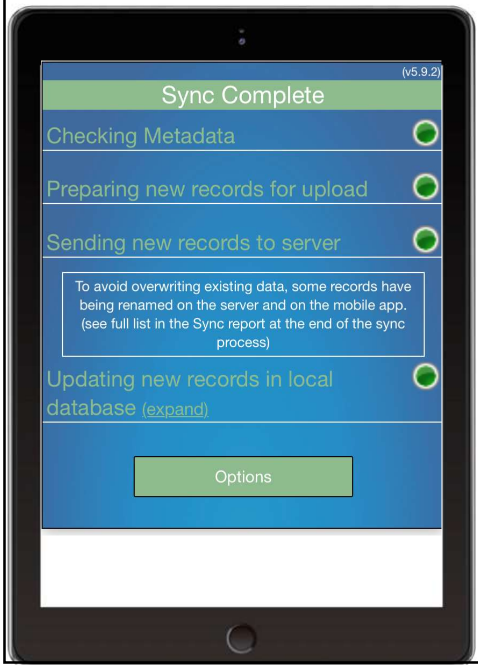

- If all control indicators light up green, the data transfer to the system was successful

digiDEM BAYERN

Anmeldung | Select project | New participant | Save data | **Data transfer** | Important terms

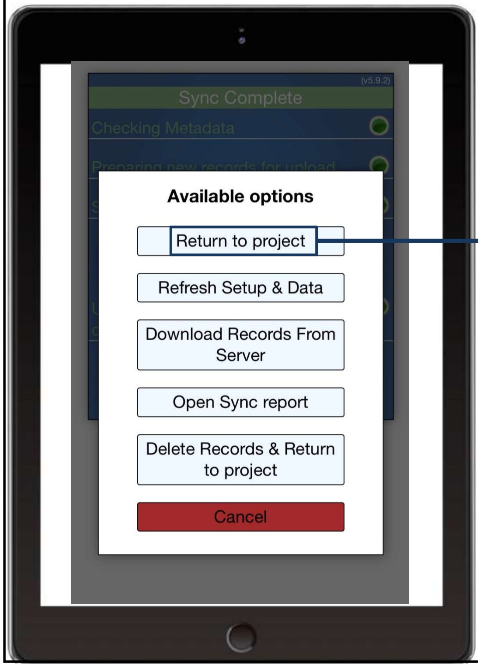

If the data transfer was successful, you can return to the project

digiDEM BAYERN

Questions?

digiDEM BAYERN

Terms from the table of content:

Terms	Meaning
My Projects	Meine Projekte
Set up Mobile Projects	Konfiguration Mobile Projekte
Change my password	Passwort ändern
About the REDCap Mobile App	Über REDCap
Updates History	Aktualisierungen
Frequently Asked Questions	Häufig gestellte Fragen
Translate the Interface (Translate)	Benutzeroberfläche übersetzen
Purpose	Zweck
Report a Bug	Fehlermeldung

digiDEM BAYERN

More terms:

Terms	Meaning
Incomplete	Unvollständig
Unverified	Ungeprüft
Complete	Vollständig
Save & Exit Form	Speichern & Erhebungsbogen schließen
Save & Stay	Speichern & bei diesem Erhebungsbogen bleiben
Show more save options	Weitere Speicheroptionen anzeigen
Delete data for this form	Daten für diesen Erhebungsbogen löschen
Cancel	Abbrechen

digiDEM BAYERN
