# Supplementary material for: Usability Evaluation of an Offline Electronic Data Capture App in a Prospective Multicenter Dementia Registry (digiDEM Bayern): Mixed Method Study
Source: JMIR Form Res. 2021 Nov 3;5(11):e31649. doi: 10.2196/31649 (PMC8600440; doi:10.2196/31649)
Supplement: Multimedia Appendix 4 [file formative_v5i11e31649_app4.pdf]

Supplementary Appendix 4

Online questionnaire in SoSci Survey

Content:

- About the participant
- System Usability Score (SUS)
- Technology Acceptance

soSci oFb - der onlineFragebogen

0% ausgefüllt

Dear Sir or Madam,

as part of a master's thesis, the usability and acceptance of the data collection method for register data using the REDCap application on mobile devices is to be investigated in the "digiDEM Bayern" project.

With your participation in this online survey you can support this.

The survey is anonymous and takes about 10 minutes. The results of this survey will be scientifically evaluated.

Thank you very much for your support!

Weiter

soSci oFb - der onlineFragebogen

20% ausgefüllt

Declaration of consent:

Voluntariness. Your participation in this study is voluntary. You are free to discontinue your participation at any time during this study without penalty.

Anonymity Your data is of course confidential, will only be evaluated in anonymous form and will not be passed on to third parties. Demographic data such as age or gender do not allow any clear conclusion about your person.

Questions. If you have any further questions about this study, you will find an imprint with contact details of the study leaders below.

I agree to the data protection declaration of consent and would like to participate voluntarily in this online survey.

- ◯ No (not participate in the study)
- ◯ Yes

Weiter

soSci
oFb - der onlineFragebogen

40% ausgefüllt

1. How old are you?

- ◯ 18-24 years
- ◯ 25-34 years
- ◯ 35-44 years
- ◯ 45-59 years
- ◯ 60 and older

2. Please enter your gender

- ◯ female
- ◯ male
- ◯ divers

3. Which of the following devices do you use (personally or professionally)?

Multiple answers possible

- ☐ Smartphone
- ☐ Tablet
- ☐ Desktop computer
- ☐ Laptop
- ☐ none

Weiter

soSci oFb - der onlineFragebogen

60% ausgefüllt

4. Questions on the system usability

Note:

The following statements refer to the usability of REDCap on mobile devices.

	strongly disagree	rather disagree	undecided	rather agree	strongly agree
**I think that I would like to use the system (REDCap) frequently.**	○	○	○	○	○
**I found the system (REDCap) unnecessarily complex.**	○	○	○	○	○
**I thought the system (REDCap) was easy to use.**	○	○	○	○	○
**I think that I would need the support of a technical person to be able to use this system (REDCap).**	○	○	○	○	○
**I found the various functions in this system (REDCap) were well integrated.**	○	○	○	○	○
**I thought there was too much inconsistency in this system (REDCap).**	○	○	○	○	○
**I would imagine that most people would learn to use this system (REDCap) very quickly.**	○	○	○	○	○
**I found the system (REDCap) very cumbersome to use.**	○	○	○	○	○
**I felt very confident using the system (REDCap).**	○	○	○	○	○
**I needed to learn a lot of things before I could get going with this system (REDCap).**	○	○	○	○	○

Weiter

soSci oFb - der onlineFragebogen

80% ausgefüllt

5. Questions on the technology acceptance

Note:

Mobile devices are understood to mean tablets and smartphones.

Perceived ease of use	strongly disagree	rather disagree	undecided	rather agree	strongly agree
**I find it easy to use mobile devices.**	○	○	○	○	○
**The interaction with mobile devices is clear and understandable for me.**	○	○	○	○	○
**I find the navigation of mobile devices easy.**	○	○	○	○	○
**I can easily remember how to perform tasks with a mobile device..**	○	○	○	○	○

Perceived usefulness	strongly disagree	rather disagree	undecided	rather agree	strongly agree
**Mobile devices are useful for my job.**	○	○	○	○	○
**Mobile devices allow me to accomplished my tasks more quickly.**	○	○	○	○	○
**The use of mobile devices makes it easier to do my job.**	○	○	○	○	○

Social influence	strongly disagree	rather disagree	undecided	rather agree	strongly agree
**Colleagues recommend that I should use mobile devices.**	○	○	○	○	○
**Supervisors at work support the use of mobile devices.**	○	○	○	○	○

Facilitating conditions	strongly disagree	rather disagree	undecided	rather agree	strongly agree
**I have the necessary resources to use mobile devices.**	○	○	○	○	○
**I have knowledge in the use of mobile devices.**	○	○	○	○	○
**I am able to use mobile devices at work.**	○	○	○	○	○

Anxiety	strongly disagree	rather disagree	undecided	rather agree	strongly agree
**I have concerns about the use of mobile devices at work.**	○	○	○	○	○
**Inappropriate exploitation of mobile devices can lead to information loss.**	○	○	○	○	○
**I hesitate to use mobile devices for fear of making mistakes I cannot correct.**	○	○	○	○	○

Weiter

soSci
oFb - der onlineFragebogen

Thank you for your participation!

We would like to thank you very much for your support.

Your answers have been saved, you can now close the browser window.
