# Supplementary material for: Usability Evaluation of an Offline Electronic Data Capture App in a Prospective Multicenter Dementia Registry (digiDEM Bayern): Mixed Method Study
Source: JMIR Form Res. 2021 Nov 3;5(11):e31649. doi: 10.2196/31649 (PMC8600440; doi:10.2196/31649)
Supplement: Multimedia Appendix 5 [file formative_v5i11e31649_app5.pdf]

Supplementary Appendix 5

Thinking Aloud results: Encoded Segments

Content:

- Usability problems
- Functionality problems
- Suggestions for improvement
- Positive aspects

Note: The research partners' statements were not translated into English because much colloquial language was used, and an English translation would possibly distort the meaning.

Usability Problems

Colour	Group	Research Partner	Code	Weighting	Segment
●	language	Transcript Research Partner 1	Usage Problems	3	Jetzt muss ich mal kurz überlegen, wie ich das hinkriege, weil es alles auf Englisch ist.
●	perceived offer character	Transcript Research Partner 1	Usage Problems	2	Also ich bin jetzt auf t0 und jetzt bin ich mir unsicher, was ich machen soll
●	knowledge error	Transcript Research Partner 1	Usage Problems	1	Ja da bin ich mir jetzt auch unsicher, weil das stimmt jetzt nicht mit den, mit den Begrifflichkeiten überein. Weil ich würde jetzt eigentlich 'incomplete' andrücken, weil es ja eigentlich nicht sein kann, aber jetzt steht hier 'Field comments', 'Pin field', 'Full screen' und 'Reset'
●	knowledge error	Transcript Research Partner 1	Usage Problems	1	Ja das kapiere ich jetzt auch nicht so ganz, weil ich habe eigentlich gedacht, dass das jetzt speichert und da stand jetzt auch dieses grüne Häkchen, dass es das gespeichert hat.
●	language	Transcript Research Partner 1	Usage Problems	3	Jetzt finde ich es mit dem Englisch immer ein bisschen schwierig, obwohl eigentlich mein Englisch nicht schlecht ist, dachte ich.
●	perceived offer character	Transcript Research Partner 1	Usage Problems	2	hier finde ich es dann schon übersichtlich das geht, aber davor war ich mir kurz ein bisschen unsicher, wo drücke ich da jetzt drauf.
●	knowledge error	Transcript Research Partner 1	Usage Problems	1	Also ich versuche jetzt einfach - ich weiß jetzt gerade nicht, wie ich da hinkomme, dass ich die 't1' praktisch mache. Probiere ich mal ein bisschen rum.
●	inconsistent interaction design	Transcript Research Partner 1	Usage Problems	2	So das kann ich jetzt nicht so eingeben, wie ich mir das vorstelle. 12.
●	language	Transcript Research Partner 2	Usage Problems	3	Da dass es halt auf Englisch ist, dass ist einfach echt blöd. Also ich glaube, ich merke es ja jetzt schon bei mir, und ich bin schon auch vom Studium her, musste ich auch manchmal englische Texte lesen. Also ich bin da jetzt nicht so ganz fremd, aber wenn halt jemand, der wirklich langjährig schon in dem Bereich tätig ist und dann keine Erfahrung hat mit der Sprache hat - ja.
●	navigation	Transcript Research Partner 2	Usage Problems	1	Ja und dann 'Logout' war einfach ganz oft zurück und dann kommt Logout. Ok ja finde ich auch nicht so schön gelöst, dass man dann zurück muss. Weil dann hat man automatisch wieder dieses 'Zurück' ist immer so - also habe ich von Word so, dass man das dann was löscht. Wäre jetzt meine Meinung dazu.
●	knowledge error	Transcript Research Partner 4	Usage Problems	1	Jetzt ist natürlich die Frage, wie ich das mache. Sie haben ja eigentlich - ja wie viele eigene Kinder haben Sie steht da und Kinder leben gemeinsam. Also da gibt es nichts, wo ich es jetzt notieren kann, dass die Frau noch ein Kind hat.
●	perceived offer character	Transcript Research Partner 4	Usage Problems	2	Genau dann gehe ich auf 'My Projects'. (4 Sek.) Ok. da fehlt der Button oder - da habe ich jetzt echt ein Blackout. Ah ok ja klar.
●	language	Transcript Research Partner 4	Usage Problems	3	Also was mir jetzt da auffällt, ist einfach, dass es Englisch ist. Ich habe zwar in der Schule Englisch gehabt, aber es ist nicht geläufig. Also da wäre es aufjedenfall gut, das hier auf Deutsch umzubenennen
●	language	Transcript Research Partner 4	Usage Problems	3	Ja, das ist bisschen gewöhnungsbedürftig. Wie gesagt, es wechselt auch ständig Deutsch, Englisch.
●	language	Transcript Research Partner 4	Usage Problems	3	Weil ich als Nutzer natürlich, ich muss schauen, dass ich meine Zeit auch gut einteile. Wenn ich dann ständig überlegen muss, was heißt das jetzt wieder.
●	knowledge error	Transcript Research Partner 4	Usage Problems	1	Ja genau, aber wo sehe ich jetzt, dass es die eins ist?
●	inconsistent interaction design	Transcript Research Partner 6	Usage Problems	2	Da haben wir wieder unseren Fehler mit der Mehrfachantwort, dass wir die geben können.
●	knowledge error	Transcript Research Partner 6	Usage Problems	1	Wie sehe ich jetzt was meine Befragung war?
●	inconsistent interaction design	Transcript Research Partner 6	Usage Problems	2	Ok 'select date'. Ja also ist sehr unübersichtlich. Ich habe nämlich eigentlich - ich wollte es reinschreiben und wollte dann sagen, warum macht es nicht einfach das Rad zum auswählen
●	language	Transcript Research Partner 7	Usage Problems	3	Ja wie gesagt, das ist noch ein bisschen verwirrend. Ein paar Mal ist es auf deutsch, dann wieder auf englisch. Da muss man schnell umschalten.
●	perceived offer character	Transcript Research Partner 8	Usage Problems	2	Da muss ich jetzt ein bisschen warten bis er es geladen hat quasi oder habe ich schon den ersten Fehler gemacht?"
●	feedback	Transcript Research Partner 8	Usage Problems	3	So für mich ist es leider nicht ersichtlich, ob die Daten übermittelt worden sind oder nicht. Das wäre für mich jetzt ein Thema.
●	navigation	Transcript Research Partner 8	Usage Problems	1	hier hätte ich jetzt das Problem, wo kann ich auf einen vorhandenen Datensatz zugreifen?
●	feedback	Transcript Research Partner 9	Usage Problems	3	Was mich jetzt ein bisschen - ich bin mir halt nicht sicher, ob das Programm nochmal den Fragebogen gecheckt hat oder ob der Fehler, den ich vorhin eventuell gemacht habe, der da noch rot aufgeleuchtet hat, ob der immer noch drin ist. Würde ich mir jetzt halt danach nochmal anschauen.
●	navigation	Transcript Research Partner 9	Usage Problems	1	Ok, ja also da war ich jetzt irgendwie bei diesem Punkt wo diese ganzen - für mich einfach nur grün und abgehakt und die einzige Möglichkeit, die ich wählen konnte war 'Options' und da hat es mich jetzt irgendwie verloren.
●	language	Transcript Research Partner 9	Usage Problems	3	Es ist ja irgendwie häufig so, dass diese englischen Subtexte für irgendwelche Programmiersprachen gedacht sind und das Deutsche für den Verbraucher und deswegen glaube ich, geht man da schneller darüber. Deswegen habe ich da vielleicht Informationen verpasst.
●	feedback	Transcript Research Partner 11	Usage Problems	3	Also hier weiß ich jetzt nicht wie ich weitermache. Wenn ich es jetzt ausschalte, ob es dann gespeichert ist - also ich meine, ich habe ja nach jedem Datensatz ja auf speichern gemacht.
●	feedback	Transcript Research Partner 11	Usage Problems	3	Wo sehe ich jetzt, dass es übermittelt ist? Ich meine es war jetzt alles grün, aber kann man da irgendwo noch nachschauen, dass es jetzt auch tatsächlich ist.
●	navigation	Transcript Research Partner 10	Usage Problems	1	Das heißt dann - jetzt kommt schon mal die Frage, probiere ich es mit 'Return'. So dann habe ich schon mal ein Problem, dass er nichts macht. Jetzt denke ich mal keine Ahnung, würde ich mal ein wenig - ok jetzt fragt er mich 'Field Comments', das heißt ich kann da, wenn ich da jetzt zum Beispiel drauf gehe - ok ist voll kacke gerade für mich. So soll ich das ungefähr sagen. Also ich bin da jetzt darauf gegangen, weil ich eigentlich weiter drücken wollte und jetzt kommt irgendwas, was zwar irgendwie mit dem Test zu tun hat, aber ich gerade im Moment null Komma null Ahnung habe. Das heißt ich würde jetzt auf 'zurück' gehen, aber jetzt fragt er mich was ich machen soll. Ich möchte ja eigentlich bleiben. So, keine Ahnung jetzt bin ich an einem Punkt wo ich sage, weiß ich nicht mehr weiter.
●	knowledge error	Transcript Research Partner 10	Usage Problems	1	Vollständig, Daten überprüft - im Moment steht ja dreimal unvollständig da, wenn ich das richtig sehe. Das ist ja richtig fucking. Das ist doch scheiße, dann habe ich ja dreimal etwas falsch gemacht. So. Dann würde ich sagen, schließen wir das ganze ab und ich gehe - nein. (lacht) Jetzt müssen wir mal schauen. Gut, dann hätte ich jetzt als erstes - dann würde ich versuchen wieder zurück zu gehen
●	perceived offer character	Transcript Research Partner 10	Usage Problems	2	Also ich habe es auf jeden Fall einmal übersehen.
●	navigation	Transcript Research Partner 10	Usage Problems	1	Eigentlich muss ich doch zum Ausloggen nichts machen oder? Also ich würde jetzt soweit zurückgehen bis ich irgendwann merke, dass es soweit ist. Also so würde ich das jetzt machen. Ich würde jetzt soweit zurückgehen bis ich wieder von vorne anfangen kann oder laden kann dann später.
●	navigation	Transcript Research Partner 12	Usage Problems	1	Wahrscheinlich ausloggen über „Managelogs“. Ich drücke das mal. (10 Sek.) Ok also ich würde es abspeichern mit 'Save to device', damit das hier im Gerät sozusagen, weil wir sind ja offline, damit es im Gerät da ist. (8 Sek.) Ok jetzt muss ich mich irgendwie noch ausloggen.
●	navigation	Transcript Research Partner 3	Usage Problems	1	Für die Testperson ist das Ausloggen aus der App kein intuitiver Vorgang. Erst durch mehrmaliges tippen auf den Zurück-Pfeil erscheint das Logout-Feld.
●	perceived offer character	Transcript Research Partner 3	Usage Problems	2	Darüber gelangt sie zur Übersicht der Befragungsprojekte. Um ein Befragungsprojekt auszuwählen, muss aktiv auf dieses getippt werden.
●	navigation	Transcript Research Partner 3	Usage Problems	1	mehrere offene Fragen nacheinander abgefragt. Um die Antwort eingeben zu können, muss die Testperson jedes einzelne Textfeld antippen.
●	inconsistent interaction design	Transcript Research Partner 3	Usage Problems	2	Manche Textfelder erfordern eine reine Zahleneingabe. Möchte die Testperson die Zahlen in das Textfeld eingeben, so öffnet sich zunächst die Qwertz-Tastatur des Tablets und eine aktive Umstellung der Tastatur auf den Ziffernblock muss durch die Testperson erfolgen.
●	inconsistent interaction design	Transcript Research Partner 3	Usage Problems	2	Die Testperson möchte ein Datum über das Datumsfeld auswählen. Durch tippen auf das Icon öffnet sich ein Feld zum scrollen bis das gewünschte Datum ausgewählt ist. Durch Antippen auf die Zeile in die das Datum eingetragen werden muss, wird das Datum nicht hinterlegt. Erst durch ein tippen neben das Icon, wird die Zeile mit dem Datum ausgefüllt.
●	feedback	Transcript Research Partner 3	Usage Problems	3	Die Testperson möchte ihre offline erhobenen Daten an den Server senden. Beim Tippen auf „Senden beginnen“ startet die Datenübertragung und es folgt eine Übersicht auf Englisch mit grün leuchtenden Kontrollleuchten im Full-Screen. Danach ist für die Testperson unklar, ob die Datenübertragung erfolgreich war und wie sie zum Befragungsprojekt zurückgelangt.
●	language	Transcript Research Partner 5	Usage Problems	3	Habe ich vorher schon erwähnt, das ist schön Englisch. I speak english very well, bröckelweis und gar nicht schnell .
●	perceived offer character	Transcript Research Partner 5	Usage Problems	2	Ok jetzt muss ich - nicht verbunden, meine Projekte. Ich glaube ich muss es jetzt anklicken.
●	language	Transcript Research Partner 5	Usage Problems	3	Oh wieder Englisch. Da bräuchte ich jetzt schon professionelle Hilfe. Da ich das nicht lesen kann.
●	knowledge error	Transcript Research Partner 5	Usage Problems	1	Wie kann ich das jetzt da eingeben. Ich glaube es ist der, oder. Oh schon wieder nichts, wo ist die drei. Siehst jetzt geht es schon los .
●	navigation	Transcript Research Partner 5	Usage Problems	1	Und wie komme ich jetzt wieder raus? Da? Nein. Ich muss auf das Ding drücken . Ach herrje, jetzt haben wir es
●	language	Transcript Research Partner 5	Usage Problems	3	Ah ja oder auch nicht. 'Incomplete' wenn man jetzt wieder Englisch könnte, dann wäre man gescheiter .
●	navigation	Transcript Research Partner 5	Usage Problems	1	So und jetzt will ich wieder rausgehen, dann gehe ich auf 'Reset' oder? Nein. So .
●	perceived offer character	Transcript Research Partner 5	Usage Problems	2	Ok. Jetzt warte ich kurz. (5 sec.) Dann drücken wir mal drauf .
●	inconsistent interaction design	Transcript Research Partner 5	Usage Problems	2	Der 12.Juni,ok. Da tut sich nichts. Tag, Monat, Jahr. (6 Sek.) Ich verstehe das nicht
●	knowledge error	Transcript Research Partner 5	Usage Problems	1	Ich wollte zuerst eine Zahl nehmen, aber dann habe ich es geschrieben. Dann gehen wir jetzt nochmal auf komplett. Ja
●	feedback	Transcript Research Partner 5	Usage Problems	3	Einmal habe ich da geschrieben, das macht er jetzt rot. (6 Sek.) Warum macht er es jetzt rot?

Functionality Problems

Colour	Research Partner	Code	Weighting	Segment
●	Transcript Research Partner 1	Functional Problems	2	Aber wenn ich jetzt auf diesen Pfeil da gehe, dann wird mir angezeigt 'Leave without saving' und dann wäre ich jetzt auch wieder verwirrt als Nutzer.
●	Transcript Research Partner 6	Functional Problems	3	Halt 'Sending modified records to server' - ok, dann schauen wir halt einmal, was nicht stimmt. "Sollte das nicht passieren?"
●	Transcript Research Partner 8	Functional Problems	2	Und hier habe ich schon mein Problem. Wie kommt man denn zu den Zahlen?
●	Transcript Research Partner 8	Functional Problems	1	Das springt jetzt wieder zurück. Da hätte ich jetzt ein Problem, da geht es wieder zurück zur eingetragenen Ehe und zur Partnerschaft zu Vorfrage.
●	Transcript Research Partner 8	Functional Problems	1	Hier habe ich jetzt ein Thema - er springt dann wieder zurück, aber wenn ich hier jetzt meinetwegen die Tastatur schließen will dann fährt er das komplette Dokument nach unten.
●	Transcript Research Partner 11	Functional Problems	2	'Error' sagt er jetzt. Ah weil das einmal 10 Nächte das will er nicht, das akzeptiert er nicht. Deswegen hat es jetzt diese - ok. Jetzt ist halt die Frage ich habe 'mal' ausgeschrieben, ob er das annimmt oder ob ich ein 'x' mache. Schauen wir mal, ob er das will. Nein. Also da weiß ich jetzt nicht, ich kann das jetzt x-Mal ausprobieren und das nächste Mal weiß ich es halt. Ich kann jetzt einmal schreiben, ob er das nimmt. Auch nicht. Also nur eins. Das heißt, der will nur Zahlen.
●	Transcript Research Partner 11	Functional Problems	2	Bei der nächsten Frage bin ich jetzt automatisch auf 'ja' gekommen, also ich würde es jetzt stehen lassen je nachdem was er antwortet und dann das andere würde ich stehen lassen, weil ich kann es jetzt nicht löschen.
●	Transcript Research Partner 10	Functional Problems	3	Aber für mich, weiß ich jetzt nicht, bräuchte ich vielleicht trotzdem eine Warnung, dass wenn ich jetzt weitermache mit dem Datensatz zwei, weiß ich gar nicht, ob ich weitermachen könnte, wenn ich weiter gemacht hätte.
●	Transcript Research Partner 5	Functional Problems	3	Ok dann starten wir mal. Das ist hier wieder schön Englisch. Halt jetzt ist eins gelb . - Datenübertragung abgebrochen

Suggestions for Improvement

Colour	Group	Research Partner	Code	Segment
●	comment / note field	Transcript Research Partner 1	Suggestions for Improvement	Ok, ich soll ja immer sagen was ich denke, das finde ich jetzt ein wenig blöd, weil da komme ich etwas durcheinander. Also ich persönlich. Und da bin ich mir auch unsicher, wie ich das am besten vermerke, weil er hat ja gesagt vier und ja.
●	instructions	Transcript Research Partner 1	Suggestions for Improvement	Dann könnt ich vielleicht nochmal, dass ich auf 'Incomplete' drücke oder so. Ja aber, dass ist ein bisschen unübersichtlich. Da würde ich es jetzt einfach so übernehmen, aber ich denke, wenn man auch die Erklärung dazu nicht hat da ist man sich dann total unsicher.
●	comment / note field	Transcript Research Partner 1	Suggestions for Improvement	Incomplete', aber da kann ich die Fragen nicht einzeln auswählen oder nicht? Und das finde ich doof. Also, dass man - oder auch einzeln dann vielleicht noch ein Kommentar dazu machen könnte, genau.
●	feedback	Transcript Research Partner 1	Suggestions for Improvement	Also da dachte ich beispielsweise, dass man da vielleicht nochmal auf einen Haken klickt oder so.
●	language	Transcript Research Partner 1	Suggestions for Improvement	Und was ich mir auch noch denke, vielleicht wär es besser das auf Deutsch zu machen die Begrifflichkeiten.
●	design	Transcript Research Partner 2	Suggestions for Improvement	Das Einzige, was mir jetzt gleich auffällt bei dem ersten war es ein Kreis und ausgefüllt und unten ist es dann so ein Haken. Ist jetzt an sich nicht schlimm, aber ich glaube es würde mehr Sicherheit geben, wenn das von der Symbolik her gleich wäre.
●	comment / note field	Transcript Research Partner 4	Suggestions for Improvement	Wobei ich da vielleicht noch, würde ich eine freie Notiz noch vielleicht reinschreiben würde, warum man denn dann annimmt, dass man eine Demenz hat. Weil das kann ja unterschiedlich sein, manchmal merkt man es selbst, manchmal merkt man es nicht. Die Frau merkt es dann.
●	perceived offer character	Transcript Research Partner 4	Suggestions for Improvement	Also da wäre es besser wenn man hinten ein Logout hätte und nicht wieder zurück müsste.
●	perceived offer character	Transcript Research Partner 4	Suggestions for Improvement	Also da wäre dann trotzdem ein Button 'Zurück' oder 'Zurück zum Hauptmenü', das wäre wirklich wichtig dann.
●	language	Transcript Research Partner 4	Suggestions for Improvement	Das ist wirklich vielleicht in einem bleiben und vielleicht auch ein Button, wär Englisch mag ist ja ok, aber dass man es vielleicht auch verändern kann. Vor allen Dingen ich habe auch Kolleginnen, die zwar deutsch sprechen, aber von einem anderen Land kommen. Da ist vielleicht dann auch nochmal eine andere Übersetzung vielleicht notwendig.
●	perceived offer character	Transcript Research Partner 4	Suggestions for Improvement	Dann drücken wir mal auf den Kalender. Also das Eingeben wäre hier praktischer. Ja genau eingeben wäre praktischer.
●	design	Transcript Research Partner 6	Suggestions for Improvement	Ich finde das 'Hiermit bestätige ich' könnte vielleicht von der Farbgebung noch ein bisschen besser rauskommen, weil das hätte ich jetzt fast überscrollt.
●	design	Transcript Research Partner 6	Suggestions for Improvement	Das könnte hier auch farblich ein bisschen besser rauskommen, würde ich sagen. Ich habe jetzt die mittlere Reife angewählt und sehe eigentlich farblich bis auf ein bisschen veränderten Punkt vorne null Unterschied zu den anderen Sachen.
●	design	Transcript Research Partner 6	Suggestions for Improvement	Ja immer wieder das Gleiche, also ich finde einfach das sollte besser rauskommen.
●	design	Transcript Research Partner 6	Suggestions for Improvement	Könnte man vielleicht - weil mir ist es jetzt gleich aufgefallen, dass die Frage aufpoppt, wenn man 'ja' ankreuzt, aber man könnte so eine Pop-up Frage auch nochmal anders hervorheben. Ich weiß nicht, ob es viel bringt, aber ich finde immer groß schreiben, bunt und so weiter, das kommt immer gut auf jeden Fall.
●	design	Transcript Research Partner 6	Suggestions for Improvement	Ich muss sowieso sagen, ich finde im allgemeinen das Erscheinungsbild von der App in Ordnung, aber man könnte noch ein bisschen daran arbeiten.
●	design	Transcript Research Partner 6	Suggestions for Improvement	Das muss alles aber ein bisschen mehr rauskommen.
●	perceived offer character	Transcript Research Partner 6	Suggestions for Improvement	So dann wollen wir uns noch aus der App ausloggen. Da muss ich ganz einfach sagen, warum nicht einfach ein Logout. Also das ist - ich denke ein Button, ich weiß ja nicht wie ihr die REDCap App an sich bearbeiten könnt, aber dass man einfach sagt, vielleicht immer rechts unten oder so einen Button worüber man immer schnell ausmachen kann
●	perceived offer character	Transcript Research Partner 6	Suggestions for Improvement	es kommt dann auch, aber ich denke einfach ein richtigen Button Datum - ja 'select date', aber dass nicht bloß steht, sondern ein Button ist auf jeden Fall.
●	feedback	Transcript Research Partner 6	Suggestions for Improvement	Ja dann ist es auch wieder schwierig. Ich meine bestätigen sollte man zumindest haben, wir haben hier nur 'clear'. Er nimmt es zwar an, aber normal ist man es gewöhnt, dass man einen Button hat.
●	design	Transcript Research Partner 6	Suggestions for Improvement	Das ist alles ein bisschen minimalistisch gehalten, wobei ich bin halt da so ein Freund für viel Farbe und alles. Vielleicht lenkt es einen anderen bloß ab. Also da würde ich sagen, ist vielleicht eine subjektive Ansicht.
●	comment / note field	Transcript Research Partner 7	Suggestions for Improvement	Also mir zeigt es jetzt eine Warnung an über die Anzahl der Kinder. Da wäre es vielleicht nicht schlecht, dass man dann sagen könnte da kann man noch ein Text darunter schreiben, wieso, weshalb, warum. Weil da kann ich ja jetzt sonst nichts machen.
●	comment / note field	Transcript Research Partner 7	Suggestions for Improvement	Also da wäre es nicht schlecht, wenn man noch zusätzlich etwas schreiben könnte bei Facharzt, würde ich jetzt sagen. Damit man festhalten kann, was das jetzt genau wäre.
●	feedback	Transcript Research Partner 8	Suggestions for Improvement	Für mich persönlich wäre es jetzt schon hilfreich, dass es dann quasi in das nächste Kästchen springt, weil dann weiß ich, dass ich hier mit der Bearbeitung fertig bin. Für mich persönlich wäre es auch einfach, wenn jetzt hier ein Häkchen wäre, dass ich sag so ich bin jetzt hier fertig und dann springt es quasi in das nächste. Wäre schön, wäre für mich halt einfacher
●	feedback	Transcript Research Partner 8	Suggestions for Improvement	Das es mir sagt, dass er fertig ist und dass es jetzt quasi weiterspringt. Das wär jetzt so mein Ansatz gewesen, aber das ist halt auch schwer zu programmieren wahrscheinlich.
●	comment / note field	Transcript Research Partner 8	Suggestions for Improvement	Hier wäre es halt jetzt schon, wenn ich jetzt hier beim Hausarzt, wenn ich da drauf klicke, dass ich dann diese Geschichte jetzt irgendwie - ich weiß nicht wie es dann für Sie ist, dass ich dann die Überweisung hätte mit eintragen können. Ob das dann hilfreich ist, weiß ich jetzt nicht. Aber hier kann ich jetzt so in die Richtung quasi nichts machen. Er ist ja dann im Anschluss weiter überwiesen worden.
●	feedback	Transcript Research Partner 8	Suggestions for Improvement	Einfach eine Bestätigung, dass jetzt zum Beispiel unten irgendwie ein Kästchen aufploppen würde, Daten erfolgreich übermittelt. Dass ich weiß ich kann jetzt zurück ansonsten - ich sage mal, wenn ich das ein paar Mal gemacht habe, dann geht es schon, aber jetzt so für den Anfang ist es für mich schon ein bisschen verwirrend. Oder halt dann keine Ahnung, wenn ich zeitgleich irgendwie zuhause an meinem Rechner sitze irgendwie eine Bestätigungsemail oder so. Oder meinetwegen irgendwie nur ein kurzen Ton. Irgendwie ein Signal, dass die Übermittlung abgeschlossen ist. Datenübertragung war erfolgreich oder irgendwie sowas. Das wäre echt eine tolle Geschichte
●	feedback	Transcript Research Partner 8	Suggestions for Improvement	Wie gesagt und jetzt wäre es schön, wenn das quasi irgendwie so, dass es abgeschlossen ist. Also nicht nur mit dem grünen Punkt, sehe ich zwar so, aber vielleicht würde es auch gehen, dass es dann quasi automatisch wieder zurück hüpft, wenn es abgeschlossen ist. Das wäre vielleicht auch noch eine tolle Geschichte. Weil sagen wir mal bei mir geht es jetzt, wenn ich es ein paar Mal gemacht habe, aber es gibt halt manche Menschen bzw. ich fühle mich da so irgendwie, ich fühle mich halt dann ein bisschen durcheinander. So Projekt abgeschlossen in diese Richtung, das wäre auf jeden Fall etwas Tolles
●	design	Transcript Research Partner 9	Suggestions for Improvement	Hier war ich jetzt kurz unsicher, ob ich darauf tippen muss oder auf den Punkt. Ok. Ich glaube, da lag es jetzt irgendwie an der Haptik, dass es erst nicht so richtig funktioniert hat.
●	instructions	Transcript Research Partner 9	Suggestions for Improvement	Da bin ich mir jetzt gerade nicht sicher, ob ich es nicht besser fände, wenn solche Warnungen am Ende des Interviews vielleicht nochmal kommen, weil so unterbricht es sehr mein Gesprächsfaden. Also das hat mich jetzt beim ersten Mal sehr irritiert und jetzt schaue ich es mir aber trotzdem einfach mal an.
●	instructions	Transcript Research Partner 9	Suggestions for Improvement	Gut, jetzt ist das hier so ein bisschen farblich markiert, das soll mir vermutlich zeigen, dass Internetnutzung nicht zu der dazugehörigen Frage gehört vor allem weil ich ja schon nach dem Internet gefragt habe. Hier hätte ich mir vielleicht ein bisschen gewünscht, dass da irgendwie so eine Interviewanweisung ist, dass ich die vorlesen muss und vielleicht eben auch die Antwortmöglichkeiten vorgebe, damit ich nicht zu viel selbst interpretiere.
●	comment / note field	Transcript Research Partner 9	Suggestions for Improvement	also ich würde es vielleicht als hilfreich empfinden, wenn man hier eine Möglichkeit hätte sich schnell auch mal Notizen hinzuschreiben
●	feedback	Transcript Research Partner 9	Suggestions for Improvement	also nochmal, ich glaube ich hätte da ein besseres Gefühl, wenn diese, dieses farbliche Feedback, wenn ich das nicht selbst reingesetzt hätte, sondern wenn das Programm mir sagen würde 'du hast deinen Fragebogen abgeschlossen, aber da fehlt noch etwas oder wir haben es nochmal überprüft und da sind alle Fragen logisch beantwortet', weil jetzt habe ich irgendwie das Bedürfnis alle nochmal anzuschauen
●	feedback	Transcript Research Partner 9	Suggestions for Improvement	Für mich sah das jetzt so aus als hätte ich irgendwie irgendetwas weggeklickt und wäre im Prozess verloren gegangen. Also das habe ich jetzt tatsächlich nicht mitbekommen. Da wäre jetzt irgendwie schön gewesen irgendwie 'Upload erfolgreich' oder sonst irgendwie etwas. Ich habe es jetzt auch - ich hatte jetzt auch irgendwie in Erinnerung, dass hier jetzt stehen würde, dass es hochgeladen ist. Ja das wäre vielleicht auch etwas, dass man dann am Ende nochmal sieht, jetzt ist es hochgeladen
●	perceived offer character	Transcript Research Partner 9	Suggestions for Improvement	Dann tippe ich den an. Da wäre vielleicht oder hätte es mir jetzt geholfen, wenn ich sehen könnte, dass es etwas zum antippen ist. Das ist ja manchmal - ich weiß zwar nicht genau wie, aber dass es optisch so hinterlegt ist, dass man weiß hier kann ich darauf klicken.
●	perceived offer character	Transcript Research Partner 9	Suggestions for Improvement	wobei dieses 'Now' wiederum irritiert. Das würde vermutlich das Datum auf heute setzen oder? Ich probiere es mal aus. Ja, das will ich natürlich nicht. Frage ich mich jetzt, warum das da ist, weil es vermutlich relativ selten vorkommt, dass wir genau an dem Tag erheben, an dem die Diagnose gestellt worden ist.
●	language	Transcript Research Partner 9	Suggestions for Improvement	Ja tatsächlich glaube ich ein bisschen. Gerade bei dieser Stelle, wo mir nicht klar war, dass die Daten jetzt hochgeladen sind, da hätte mir vielleicht mehr geholfen, weil man deutsche Texte einfach wesentlich schneller überfliegen oder erfassen würde. Ich glaube da wäre ich gar nicht auf hier leuchtet alles grün weiter oder bzw. ich hätte es eher verstanden, das ist für mich gedacht als Erheber.
●	comment / note field	Transcript Research Partner 11	Suggestions for Improvement	Das wäre es jetzt schön, wenn man das irgendwo reinschreiben könnte.
●	perceived offer character	Transcript Research Partner 11	Suggestions for Improvement	Ja, genau. Einfach Logout wäre deutlicher gewesen.
●	feedback	Transcript Research Partner 11	Suggestions for Improvement	Das es erfoglreich war, ja. Weil wenn ich mich darauf verlassen kann, dass wenn die Punkte grün sind, dass es auch gesendet ist. Mir geht es jetzt mehr oder weniger zu überprüfen, hat er alles übersendet. Auch wenn der grüne Button kommt mit dem Haken sehe ich ja nicht ist es jetzt wirklich - Sie sehen schon ich bin da ein bisschen skeptisch mit der Technik, weil so - Also dass ich jetzt sage, ok ich habe es jetzt gesendet.
●	perceived offer character	Transcript Research Partner 11	Suggestions for Improvement	Bei der nächsten Frage bin ich jetzt automatisch auf 'ja' gekommen, also ich würde es jetzt stehen lassen je nachdem was er antwortet und dann das andere würde ich stehen lassen, weil ich kann es jetzt nicht löschen. Also da wäre es jetzt schön, wenn ich irgendwie so eine Retoure-Taste hätte. Ja genau, weil ich bin da jetzt ausversehen darauf gekommen
●	feedback	Transcript Research Partner 10	Suggestions for Improvement	Was ich jetzt trotzdem nicht so gut finde. Also ich würde da gerne bestätigen, aber ist halt so wie es ist.<br>Weil im Moment schon, weil irgendwann muss ich ja schauen, ob alles ok ist. Wenn es jetzt zum Beispiel nicht ok wäre, dann müsste ich erstmal wieder suchen wo ist jetzt dann hier, wo habe ich vielleicht etwas falsches vielleicht eingegeben oder nicht oder wo habe ich etwas vergessen. Also das ist von meiner Anwendung jetzt, es wäre gut, wenn ich es in diesem Moment schon bestätigen würde
●	instructions	Transcript Research Partner 10	Suggestions for Improvement	Also wenn - eigentlich müsste er mir das aber gleich bringen. Das bringt er mir jetzt, zumindest jetzt.

●	feedback	Transcript Research Partner 10	Suggestions for Improvement	Dann bin ich jetzt da wieder auf der richtigen Seite. Jetzt kommt natürlich wieder, wenn ich es jetzt ausgebessert habe, jetzt habe ich eigentlich wieder den Drang dazu, dass ich irgendwo darauf drücke, dass es ok ist. Dass ich zum Beispiel auf 'Return' drücke. Ich fühle mich im Moment ein bisschen unwohl jetzt zu der nächsten Frage zu gehen
●	feedback	Transcript Research Partner 10	Suggestions for Improvement	So jetzt habe ich alle drei abgegeben. Jetzt bin ich auch verwirrt, weil ich jetzt auch wieder - was heißt verwirrt, ich möchte jetzt auch gerne wieder die in Anführungstrichen die Taste haben, wo ich jetzt die Abhakungen habe. Da weiß ich jetzt gar nicht mehr, wie man das tun muss.
●	instructions	Transcript Research Partner 10	Suggestions for Improvement	Ja, dass etwas aufploppt. Es ist ja eigentlich ganz simpel oder auch in diesem Fall logisch, aber ich sage jetzt mal in meinem Element wie ich jetzt bin, wäre es mir lieber es ploppt auf.
●	instructions	Transcript Research Partner 10	Suggestions for Improvement	Muss ich hier etwas drücken? Ah. Ja, das ist halt - das ist zum Beispiel auch etwas das verschieden ist. Ich bin einer, der ist sehr ungeduldig und überfliegt viel und dann kommt genau das raus, was jetzt gerade passiert ist. Also das ist für mich jetzt völlig normal und dass ich dann auch merke, ok da stimmt etwas nicht. Aber für mich, weiß ich jetzt nicht, bräuchte ich vielleicht trotzdem eine Warnung, dass wenn ich jetzt weitermache mit dem Datensatz zwei, weiß ich gar nicht, ob ich weitermachen könnte, wenn ich weiter gemacht hätte.
●	comment / note field	Transcript Research Partner 12	Suggestions for Improvement	ier wäre es eventuell besser, noch eine dritte Antwortmöglichkeit zu haben, wo man etwas in ein Freifeld schreiben könnte und so das zum Beispiel hinterlegen.
●	feedback	Transcript Research Partner 12	Suggestions for Improvement	Eventuell wenn man viel macht, verliert den Überblick, dann wäre es natürlich gut, wenn eine Meldung käme zum Beispiel 'Alle grün'. Ok 'Sync complete'.
●	perceived offer character	Transcript Research Partner 3	Suggestions for Improvement	Dauerhaftes Logout-Feld auf dem Screen anzeigen.
●	perceived offer character	Transcript Research Partner 3	Suggestions for Improvement	Hinweis in der Schulung geben, dass das Befragungsprojekt angetippt werden muss, um mit der Datenerhebung starten zu können.
●	feedback	Transcript Research Partner 3	Suggestions for Improvement	Nach der Eingabe einer Antwort in das Textfeld soll über eine Bestätigung der Cursor in das nächste Textfeld weitergeleitet werden, sodass ein antippen entfällt und der Gesprächsfluss nicht gestört wird.
●	perceived offer character	Transcript Research Partner 3	Suggestions for Improvement	Die Textfelder bei welchen eine Zahleneingabe erwartet wird, sollen beim Antippen den Ziffernblock der Tastatur öffnen
●	perceived offer character	Transcript Research Partner 3	Suggestions for Improvement	Kurzer Hinweis in der Schulung, wie mit dem Datumsfeld umzugehen ist.
●	instructions	Transcript Research Partner 3	Suggestions for Improvement	Allerdings ist nicht ersichtlich wie viele Erhebungsbögen bereits ausgefüllt wurden und wie viele noch bearbeitet werden müssen.<br>Empfehlung der Testperson: Anzeige des Bearbeitungsfortschrittes mittels Statusanzeige, wie zum Beispiel 18 von 20 Erhebungsbögen sind erledigt.
●	feedback	Transcript Research Partner 3	Suggestions for Improvement	Kurzer Hinweis auf Deutsch nach der Datenübertragung, ob diese erfolgreich war.
●	language	Transcript Research Partner 3	Suggestions for Improvement	Menüpunkte und Funktionen werden manchmal auf Deutsch, manchmal auf Englisch angezeigt<br>Empfehlung der Testperson: Einheitliche Übersetzung in die deutsche Sprache

Positive Aspects

Colour	Group	Research Partner	Code	Segment
●	navigation	Transcript Research Partner 1	Positive Aspects	Und jetzt komme ich auch besser rein. Am Anfang war mir das ein bisschen - wo klicke ich da jetzt hin, aber jetzt verstehe ich es schon besser.
●	feedback	Transcript Research Partner 1	Positive Aspects	Ja und jetzt ist grün also gehe ich davon aus, dass alles gespeichert ist.
●	navigation	Transcript Research Partner 2	Positive Aspects	Ok, das ging jetzt eigentlich ganz angenehm, fand ich.
●	design	Transcript Research Partner 2	Positive Aspects	Was mir auch gleich vielleicht auffällt, ich finde die Größe auch ganz gut zum Tippen. Also ich finde das ist ja auch immer wichtig
●	design	Transcript Research Partner 2	Positive Aspects	Ja das finde ich jetzt ganz gut, dass es mir das dann auch gleich anzeigt eben, dass es dann ausgefüllt ist der Kreis. Das sehe ich dann ganz gut. Das ist ja auch wichtig, wenn man dann so in der Befragung ist, dass man dann gleich sieht, ok ist abgezeichnet.
●	instructions	Transcript Research Partner 2	Positive Aspects	Ja da finde ich es jetzt auch gut, dass es dahintersteht mit der Mehrfachantwort möglich.
●	design	Transcript Research Partner 2	Positive Aspects	Aber ich finde die Darstellung jetzt angenehmer. Also ich weiß jetzt nicht, ob es generell einfach weil es sehr gekürzt wurde - aber so vom ersten Blick als jetzt die . wenn ich es über mein Laptop anschaue.
●	navigation	Transcript Research Partner 4	Positive Aspects	Das ist eigentlich gut aufgebaut.
●	structure	Transcript Research Partner 4	Positive Aspects	Das ist gut aufgelistet hier.
●	feedback	Transcript Research Partner 4	Positive Aspects	das ist gut dass es aufgezeigt wird.
●	navigation	Transcript Research Partner 6	Positive Aspects	Das ist bisher sehr übersichtilich.
●	structure	Transcript Research Partner 6	Positive Aspects	Jawohl das ist auch sehr gut gelöst, dass man mit dem Pop-up arbeiten kann.
●	feedback	Transcript Research Partner 6	Positive Aspects	Warnung: Bitte überprüfen Sie die Angabe: die Anzahl der Kinder im Haushalt und die Anzahl der Personen im Haushalt stehen im Konflikt zueinander'. Ja das ist auch schon mal sehr hilfreich auf jeden Fall, dass man da gleich die Fehlermeldung bekommt.
●	feedback	Transcript Research Partner 6	Positive Aspects	Das ist top gelöst von der App, weil wenn man so - man behält nicht alles im Kopf und ich denke, dass so etwas gerne mal untergehen kann. Finde ich sehr gut.
●	instructions	Transcript Research Partner 6	Positive Aspects	Mehrantwort möglich, das ist auch sehr schön
●	design	Transcript Research Partner 6	Positive Aspects	Jetzt wollen wir das Ganze auf 'complete' setzen. Das ist eigentlich auch total übersichtlich.
●	structure	Transcript Research Partner 6	Positive Aspects	Sehr schön das Pop-up, da kommt eine neue Frage bei 'ja'.
●	feedback	Transcript Research Partner 6	Positive Aspects	Das ist schön der grüne Haken, das mag ich.
●	navigation	Transcript Research Partner 6	Positive Aspects	Also das ist eigentlich alles übersichtlich gestaltet.
●	feedback	Transcript Research Partner 6	Positive Aspects	Ach das gefällt mir, viel mehr einbauen.
●	structure	Transcript Research Partner 7	Positive Aspects	Ja so an sich ist die App finde ich super aufgebaut.
●	learnability	Transcript Research Partner 7	Positive Aspects	Wenn man das ein paar Mal gemacht hat, dann kommt man auch eher dahinter, sage ich jetzt mal.
●	learnability	Transcript Research Partner 7	Positive Aspects	Ja, damit kann man gut arbeiten.
●	navigation	Transcript Research Partner 8	Positive Aspects	Login', das hat geklappt sehr schön.
●		Transcript Research Partner 8	Positive Aspects	Es geht sehr schnell auf.
●	learnability	Transcript Research Partner 8	Positive Aspects	Ist auch fix gegangen, haben wir gut hinbekommen.
●	learnability	Transcript Research Partner 8	Positive Aspects	Ansonsten bin ich jetzt eigentlich echt gut zurecht gekommen. Ging es ganz gut.
●	learnability	Transcript Research Partner 9	Positive Aspects	Es ging schon einmal sehr intuitiv eigentlich durch, gerade weil es auch immer irgendwie die obersten Sachen waren und man nicht lange suchen musste, um das einzuführen, also um das Richtige zu finden.
●	feedback	Transcript Research Partner 9	Positive Aspects	Dieser große, grüne Pfeil gibt mir ein gutes Gefühl.
●	instructions	Transcript Research Partner 9	Positive Aspects	Das war jetzt da wiederum schön, dass man sieht, dass diese Mehrfachantworten möglich sind.
●	design	Transcript Research Partner 9	Positive Aspects	Gut, dann setz ich das wieder auf 'complete'. Das kann man auch nicht übersehen, das habe ich nämlich am Anfang gedacht, dass es vielleicht passieren könnte.
●	design	Transcript Research Partner 9	Positive Aspects	Dann 'Daten an den Server senden', das kann man ja quasi nicht verpassen.
●	instructions	Transcript Research Partner 11	Positive Aspects	Also so vom bediene her ist es super. Es steht alles da was man zu tun hat. Also es ist ganz klar.
●	navigation	Transcript Research Partner 10	Positive Aspects	Also selbst für mich bis jetzt idiotensicher.
●	learnability	Transcript Research Partner 10	Positive Aspects	also soweit ist es für mich klar, wenn ich irgendetwas mache gerade im Moment. Es ist klar, weil da muss ich eh bloß die Zahl eingeben.
●	feedback	Transcript Research Partner 10	Positive Aspects	Das finde ich jetzt soweit ok. Das gibt mir persönlich wieder die Sache, dass ich sage jetzt habe ich das zumindest einmal erledigt und es scheint momentan für mich richtig zu sein. Ich fühle mich jetzt dabei nicht unbedingt so unwohl, dass irgendetwas nicht passen könnte. Von daher sehe ich, dass das oberste gemacht ist und jetzt gehe ich, denke ich mal, auf den zweiten Punkt.
●	instructions	Transcript Research Partner 10	Positive Aspects	Da sehe ich wieder als Erstes, dass ich nach wie vor bei der Teilnehmenden-ID Nummer 1 bin. Das ist für mich schon mal auch wieder in Anführungsstrichen beruhigend, weil wenn ich zum Beispiel die Umfage öfters machen würde, dann habe ich ja mal die 243 und dann kann ich immer für mich nochmal schauen, bin ich da richtig oder habe ich mich irgendwohin verdrückt. Das gibt mir persönlich wieder ein wenig eine Sicherheit
●	feedback	Transcript Research Partner 10	Positive Aspects	Ok im Prinzip habe ich es jetzt gemacht. Es ist alles grün. Es ist alles ok.
●	navigation	Transcript Research Partner 12	Positive Aspects	Ok, also 'Login' ging schon mal sehr gut, es ist übersichtlich und es ist gut zu machen.
●	learnability	Transcript Research Partner 12	Positive Aspects	Und an sich war es gut durchzuführen. Ich hatte natürlich auch einen sehr, sehr guten Interviewpartner, der die vorgegebenen Antworten auch schon kannte. (lacht) Aber an sich war es jetzt sehr einfach durchzuführen. Also ich bin jetzt zwar nicht der geborene Interviewer, aber ja war denke ich einfach, war gut nachzuvollziehen.
●	design	Transcript Research Partner 3	Positive Aspects	Testperson empfindet die Darstellung und das Layout in der mobilen Version (auf dem Tablet) übersichtlicher, als in der Desktop-Version.
●	learnability	Transcript Research Partner 3	Positive Aspects	Die Testperson kommt alles in allem mit der Bedienung der App zurecht. Sie spricht von einer „einfachen Handhabung".
●	learnability	Transcript Research Partner 5	Positive Aspects	Ok. Herr Maier ich denke beim zweiten Mal, wenn ich das mache, wird es vielleicht ein bisschen schneller gehen.
●	feedback	Transcript Research Partner 5	Positive Aspects	Da war jetzt auch ein grüner Punkt, jetzt gehen wir mal davon aus, dass wir gut sind. Jetzt haben wir einen grünen Punkt, dass wir es vollständig befragt haben .
●	learnability	Transcript Research Partner 5	Positive Aspects	Es ist schon viel - also ich habe so gut wie kein Handy und lebe auch frei von Internet und ist für mich schwierig und ich spreche kein Englisch. Es ist schon - wenn man es öfters macht ok.
